# Machine learning-based feature selection suggests circulating proteins as best at predicting and diagnosing tuberculosis in *Mycobacterium tuberculosis*-exposed rhesus macaques

**DOI:** 10.3389/fcimb.2026.1880496

**Published:** 2026-08-06

**Authors:** Taylor A. L. Woodard, Smriti Mehra, Deepak Kaushal, Vitaly V. Ganusov

**Affiliations:** 1 Host-Pathogen Interactions program, Texas Biomedical Research Institute, San Antonio, TX, United States; 2 Department of Mathematics, College of Science, University of Texas at San Antonio, San Antonio, TX, United States; 3 Disease Intervention and Prevention program, Texas Biomedical Research Institute, San Antonio, TX, United States

**Keywords:** *Mycobacterium tuberculosis*, biomakers, machine learning, non-human primates, tuberculosis

## Abstract

*Mycobacterium tuberculosis* (Mtb), bacteria causing tuberculosis (TB), is a leading cause of morbidity and mortality worldwide. Even though billions of individuals have evidence of past or present Mtb infection and millions develop TB yearly, most individuals exposed to Mtb do not progress to TB (active disease) and successfully control (and perhaps eliminate) the infection. Factors determining the likelihood of TB progression of Mtb-exposed individuals remains poorly understood, however. Mtb-exposed non-human primates (NHPs) such as rhesus macaques (RMs) also exhibit variable likelihood of developing active disease. Previous analysis of Mtb-exposed RMs suggested that blood proteins such as C-reactive protein (CRP), albumin to globulin (A/G), and kynurenine-to-tryptophan ratios and blood leukocytes (e.g., neutrophil frequency) were best correlated with TB diagnosis. We extended this previous work by using several machine learning (ML) techniques to further quantify the relative contribution of 20 features (and their derivatives) to predict development or diagnosis of active disease in Mtb-exposed RMs. We found that independent of the specific ML technique (random forest, linear support vector machine, or logistic regression), CRP and A/G ratio values and lung CFU were most informative at discriminating between animals with active disease or no disease; furthermore, when excluding endpoint measurements (measurements done at necropsy), CRP.peak and A/G.bottom dramatically outperformed all other features at determining progression to active disease. Top 5 (when including all features) or top 2 (when excluding endpoint measurements) features together had nearly as high discriminatory power (AUC ≥0.98) as all 20+ features. Importantly, in our analysis, frequency of blood leukocytes (e.g., percent of neutrophils or lymphocytes) or their ratios (e.g., neutrophil to lymphocyte ratio) had relatively poorer predictive or diagnostic value (AUC<0.8). Our results thus suggest that a combination of CRP and A/G ratio have a high power at predicting and/or diagnosing TB in Mtb-exposed RMs. Future studies will need to investigate if temporal changes in blood protein concentrations and leukocyte frequencies may further improve our ability to predict and/or diagnose TB in NHPs.

## Introduction

Tuberculosis (TB) is a leading cause of morbidity and mortality worldwide. According to the WHO 2024 Global Tuberculosis Report, TB remains a significant global health challenge, with an estimated 10.8 million new cases bringing the incident rate to 134 per 100, 000 population (World Health Organization, 2024). The report states that 1.25 million total deaths were attributed to TB in 2023, with 1.09 million of those deaths being in HIV-negative individuals (World Health Organization, 2024). Importantly, the same report estimates that the number of individuals with TB that are not diagnosed with the disease remains high (2.7 million in 2023) even though this diagnostic gap did decline in 3 years (from 4 million in 2020/2021) (World Health Organization, 2024). Novel methods that allow faster and more precise diagnosis individuals with TB would help to close the diagnostic gap (MacLean et al., 2019).

In addition to difficulties at diagnosing individuals with TB, identifying individuals who progress to active disease and who thus may benefit from preventive TB treatment (TPT) remains difficult (Araújo-Pereira et al., 2026). Exposure to Mtb often results in conversion of tuberculin skin test (TST) or Interferon Gamma Release Assay from negative to positive, and such conversion is a risk factor of progressing to TB (Machingaidze et al., 2012; Araújo-Pereira et al., 2026). TPT of TST/IGRA converters with a single drug such as isoniazid for 6–12 months or a combination of drugs such as INH and rifapentine for 3 months (3HP) is highly effective at preventing TB development (Ferebee, 1970; Hamada et al., 2018). However, only a small proportion of recent converters (2%-10%) would develop TB and thus would benefit from TPT (Ferebee, 1970); yet, the tools available to identify individuals at highest risk of disease progression following Mtb exposure are limited. There have been a wealth of signatures, derived from gene expression from whole blood samples, aimed at predicting individuals at highest risk of progressing to active disease (Gupta et al., 2020; Mulenga et al., 2020; Vargas et al., 2023). Interestingly, a recent clinical trial found that TPT of individuals with signature scores, predicting TB development, did not significantly reduce the overall TB rate as compared to placebo arm (Scriba et al., 2021), highlighting the need to better understand how Mtb-exposed individuals progress to TB and which of them would most benefit from preventive treatment.

Studying TB progression in humans requires long-term cohort studies which are expensive; therefore, non-human primates (NHPs) such as rhesus or cynomolgus macaques have been used to delineate factors responsible for control of Mtb or disease progression. Exposure of rhesus macaques (RMs) to Mtb leads to different outcomes often dependent on the exposure dose; for example, exposure to a low dose of CDC1551 (10 CFU/animal) results in only a minority of animals developing active disease (∼10%) whereas at exposure to higher doses of CDC1551 (100 CFU/animal), nearly all animals (∼90%) become symptomatic within 3–4 months (Sharan et al., 2021; Arora et al., 2025; Turnbull et al., 2025). Animals that become symptomatic following exposure to Mtb display cardinal features of TB such as chest X-ray abnormalities, fever, loss of appetite (and sometimes of weight), and detectable mycobacteria in the bronchial alveolar lavage (BAL) (Esaulova et al., 2021; Gough et al., 2022). In a recent study, Gough et al. (2022) summarized data from multiple experiments with Mtb-exposed RMs that either had active disease or remained asymptomatic during the observational period (typically for 16–20 weeks). The authors collected data on several blood-derived proteins such as C-reactive protein (CRP), albumin-to-globulin (A/G) ratio, kynurenine, tryptophan, and all major white blood cell subsets (neutrophils, lymphocytes, and monocytes) prior to infection, when these markers reach their peak or nadir (bottom) values, or just prior to necropsy. The authors then used a number of tests to define which of these markers are best at discriminating between active disease and asymptomatic Mtb infection (MTBI) and used linear regression to find which markers may be best predicting lung bacterial CFU. They found that CRP, A/G, and kynurenine-to-tryptophan ratios, along with neutrophil frequencies, were able to discriminate between animals with active disease vs. asymptomatic infection (Gough et al., 2022).

Here, we build on this previous study and reanalyze the nearly the same dataset (our data exclude kynurenine and tryptophan measurements) using a variety of machine learning (ML)-based techniques with the aim to identify additional markers that discriminate between active disease and asymptomatic infection. We also investigated the power of these makers to predict development of disease by excluding measurements done prior or at necropsy. Interestingly, independently of the ML-based method, blood protein measurements (CRP and A/G ratio) at their peak (CRP) or bottom (A/G ratio) values and measured at necropsy together with lung CFU were best at discriminating between animals with active disease or MTBI (AUC ≥0.95) whereas commonly cited metric such as neutrophil to lymphocyte ratio had poorer predictive power (AUC<0.8). Only CRP.peak and AG.bottom were sufficient to discriminate between animals that will progress to active disease or have active disease, and adding other metrics did not improve the predictive power of these ML-based models. Taken together, our results suggest that in Mtb-exposed RMs, blood biomarkers such as CRP and A/G ratio are highly predictive of active disease development that exceeds that of other commonly used white blood cell-based markers.

## Materials and methods

### Experimental data

**Figure 1:**
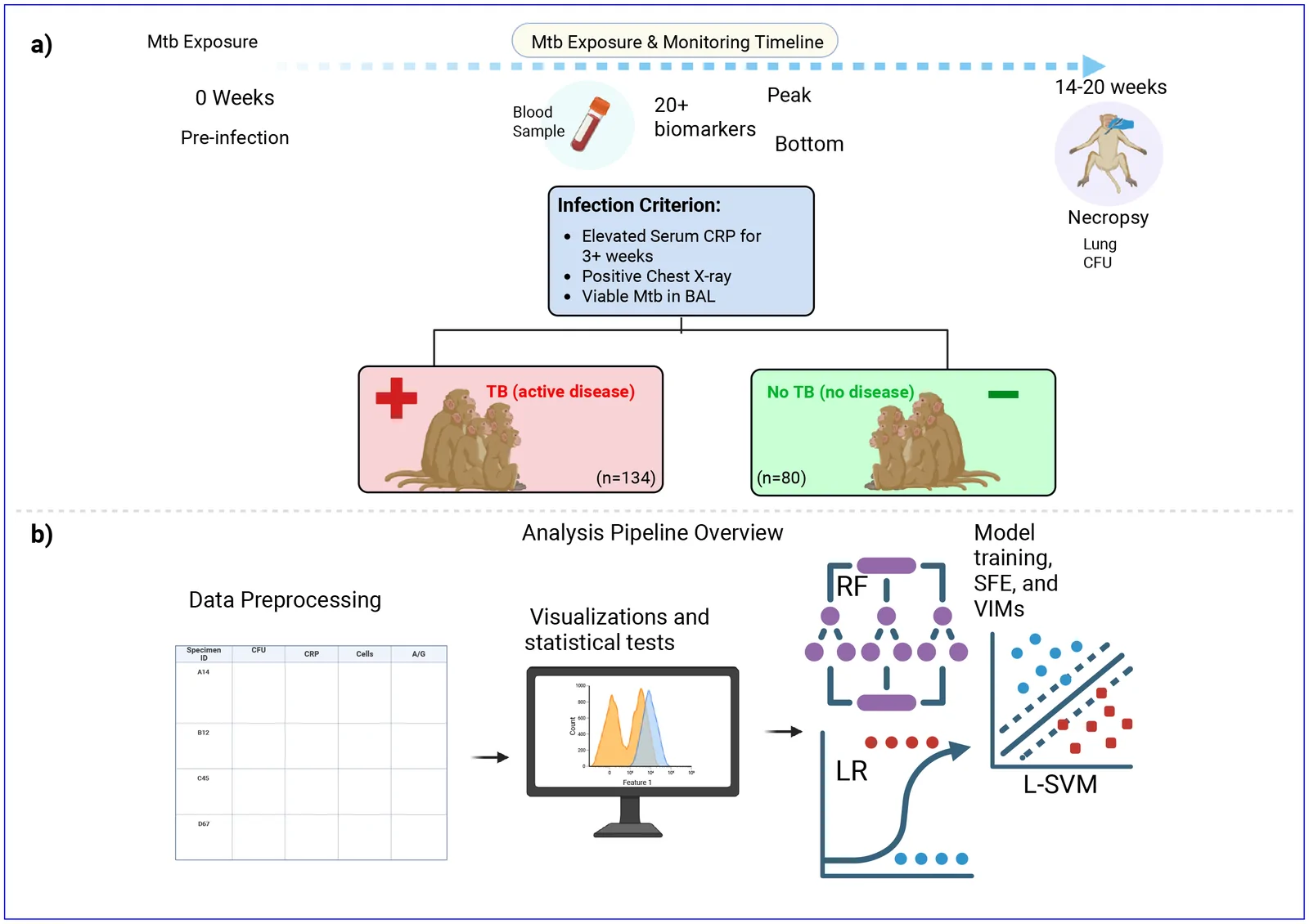
Experimental design and data analysis pipeline. **(a)** Previous study by Gough et al. (2022) summarized data from multiple experiments in which rhesus macaques were exposed to *Mycobacterium tuberculosis*, and multiple blood markers were systematically measured until necropsy (see Materials and methods for detail. By necropsy, all animals were classified as either having active disease/tuberculosis (“TB”, *n* = 134) or being asymptomatic (“no TB”, *n* = 80). Measurements of blood markers were classified into four groups, namely, preinfection, peak value, minimal value (“bottom”), and endpoint (prior to necropsy). **(b)** Our analysis pipeline consisted of traditional preprocessing and data cleaning steps. Following this, we constructed visualizations of the data to aid in our use of statistical testing. Finally, we developed and trained our models (Random Forest [RF], logistic regression [LR], and linear support vector machine [L-SVM]), performed single feature evaluation (SFE), evaluated variable importance metrics (VIMs), and studied their impact on data interpretation.

Our data come from a previous study where the authors systematically collected data on various blood biomarkers and CFU measurement at necropsy of rhesus macaques (RMs) that were exposed to variable doses of different Mtb strains (CDC1551, H37Rv, or Erdman) and followed over time (Gough et al., 2022). In total, there were data for 225 animals; all these animals were from control arms of the experiments, i.e., these animals were not coinfected with SIV and were not treated (**Figure 1A**). All animals were monitored for signs of active disease. Per Gough et al. (2022), animals were considered to have active disease if they had 1) higher than normal serum CRP levels for three weeks, 2) higher than a score of 1 on chest X-rays at one of the measurements, or 3) presence of viable Mtb bacilli in BAL. Additionally, if the Mtb-exposed animals displayed no symptoms of active disease and yet tested positive for tuberculin skin test, they were defined as animals with evidence of Mtb infection (MTBI)—a state that has been previously defined as latent TB infection (LTBI) (Coussens et al., 2024; Warner et al., 2025). Of note, recent reevaluation of TB nomenclature suggests avoiding using term “LTBI” due to its ambiguity; it is recommended replacing it with “Mycobacterium tuberculosis infection” (MTBI) to indicate individuals with evidence of present or past infection with Mtb, typically based on positive TST or IGRA test (Coussens et al., 2024; Warner et al., 2025). WHO has been also using the term TBI (TB infection) (WHO, 2021). Our dataset includes measurements for *n* = 214 animals with *n* = 134 having active disease while *n* = 80 being asymptomatic within the observational period (**Figure 1A**). This subset is smaller than the original dataset with 225 animals from Gough et al.; this is because the previous study had additional data from 11 animals with measurements of kynurenine and tryptophan that were not available in our dataset. We should also note that there were a few missing values for some features and some animals; for example, CRP.peak values were only available for 78/80 of animals with active disease (but for all 134/134 animals with no active disease).

**Figure 2:**
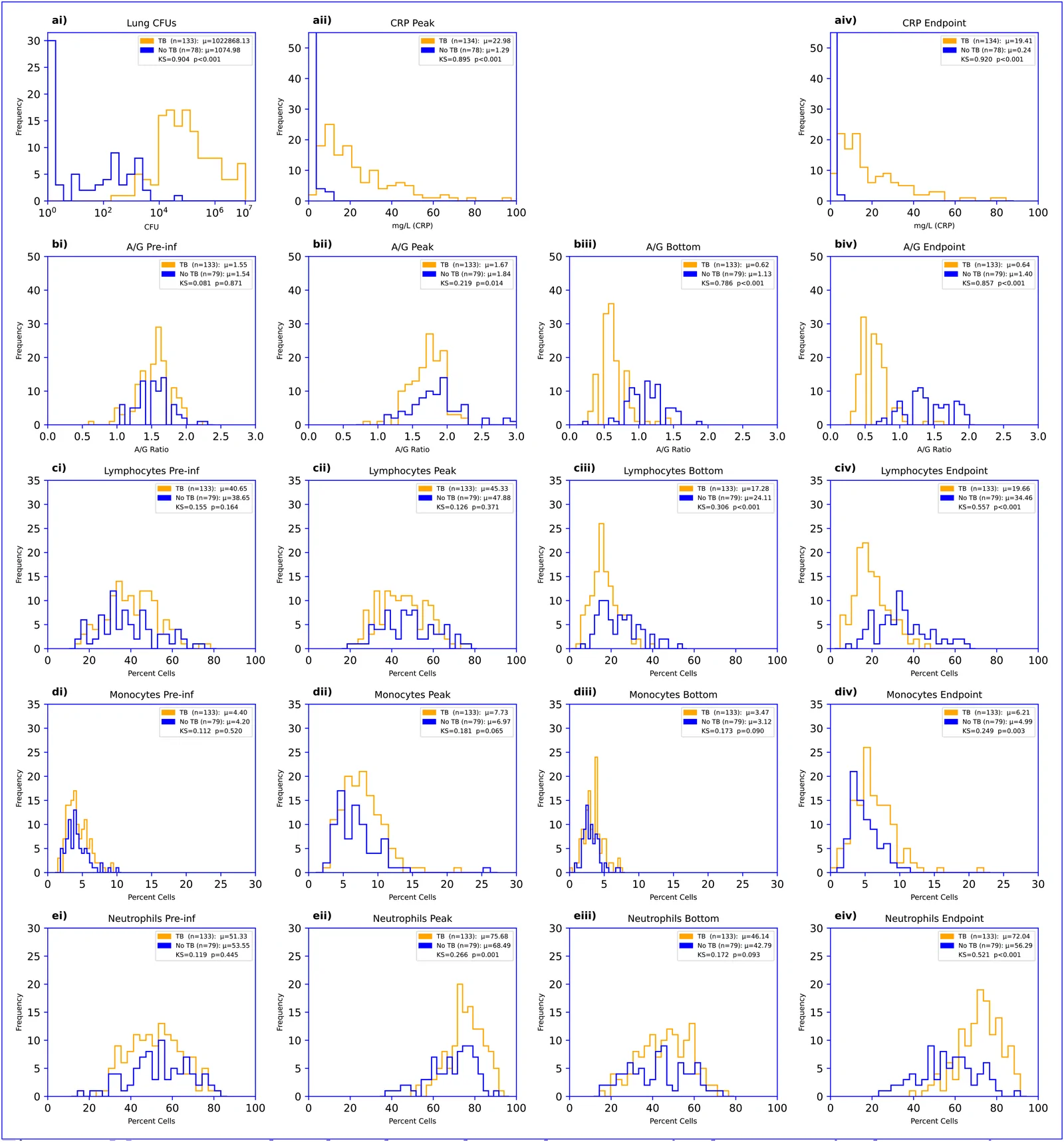
Most measured markers do not show a clear separation between animals progressing to active disease vs. asymptomatic animals. We show distribution of CFU burden in the lungs **(ai)**, Serum CRP levels (aii, iv), albumin to globulin ratios **(b)**, percent of lymphocytes **(c)**, percent of monocytes **(d)**, and percent of neutrophils **(e)** at different phases of infection (preinfection, peak, bottom, and endpoint). Note that lung CFU was measured at endpoint. Orange histograms are for animals with active disease (TB), and blue histograms are for asymptomatic animals (no TB); *μ* denotes mean values, and *D* and *p* are KS statistics and p value from the KS test (see Materials and methods for detail).

All animals were followed over time with typically weekly measurements of vital signs, blood components (cells and some proteins), and periodic BAL and chest X-rays. For each animal, there was a defined measurement of multiple blood markers (e.g., **Figure 2**; **Supplementary Table S1**) but of out of all measurements, only the following values were kept in the dataset: preinfection, peak, bottom (nadir), and endpoint (sample done prior to or at necropsy). In addition, for each of the animal, there was a measurement of lung CFU (at necropsy), thus in total resulting in 20 basic features (**Supplementary Table S1**). Following previous analysis (Gough et al., 2022), we also calculated ratios of all leukocyte subsets, e.g., neutrophil to lymphocyte (N-L) ratio. To calculate the ratios, we used values measured at a particular “stage, ” e.g., the N-L.Peak ratio was calculated as N.Peak/L.Peak. This procedure may introduce some errors; for example time when neutrophils reach their peak may not be the time when lymphocytes reach their peak (see also Discussion). In total, we had 31 features to characterize disease development in Mtb-exposed RMs.

### Statistics

We performed a number of different statistical analyses (**Figure 1B**). For most of our analyses, we used raw (untransformed data), but in some cases, we log-transformed CFU values because CFU varied orders of magnitude between animals. We used $\text{arcsin }(\sqrt{x})$ transformation for percents of cells as such transformation tends to normalize frequency data, and log (1+x) (log base 10) for other features in some analyses (see below). We characterized differences between values using t-test or F-test (**Supplementary Table S2**; **S3**) or using PCA (**Supplementary Table S4**). We used the Kolmogorov–Smirnov (KS) statistic to characterize differences in distribution of different features between animals with active disease or asymptomatic animals. We also used 1-Wasserstein distance (*D_W_*, also known as Earth Mover’s distance) computed on empirical samples in native units, which is the average amount of work observations that would need to shift in order to make the distributions match. Finally, we used Cohen’s d as standardized mean effect size or the difference in means based on the standard deviations of the feature per class.

### Machine learning

For most of our analyses, we used Python 3.14. To quantify relationships between individual features and disease status or between individual features, we used Pearson correlation (function corr in package Numpy) (Harris et al., 2020). By extending our correlation analysis, we also applied Seaborn’s ward clustering method (Waskom, 2021), implemented in package clustermap, which performs hierarchical agglomerative clustering using Ward’s linkage to group features based on similarity in correlation structure.

For machine learning, we used Random Forest (RF) Classifier, Logistic Regression (LR), and Linear Support Vector Machine (L-SVM) from Scikit Learn (Pedregosa et al., 2011). These models were selected to span a range of model classes, including ensemble-based nonlinear methods (RF) and linear classifiers (LR, L-SVM), allowing us to assess whether predictive performance depended on model complexity or feature interactions. We performed both individual feature and full feature set analyses, as this approach in machine learning allows identification of both highly informative features and those contributing minimally to predictive performance (Guyon and Elisseeff, 2003). Due to the invariant properties of the Random Forest model under monotonic transformations of input features, raw data were used directly for RF training and testing. In contrast, LR and L-SVM models are sensitive to feature scaling and distribution; therefore, we applied an $\text{arcsin }(\sqrt{x})$ transformation to cell percentage features and a log(1 + *x*) transformation to all other features to stabilize variance and reduce skewness. All features were subsequently standardized prior to model fitting.

We evaluated performance of individual models using stratified 5-fold cross-validation. In each fold, approximately 80% of the data were used for model training and the remaining 20% were held out for validation, with the held-out partition rotated across folds so that each sample was evaluated once. Stratification was performed through the StratifiedKFold object in scikit-learn, preserving the relative proportions of the outcome classes across folds. We used Receiver Operating Characteristic (ROC) analysis with area under the curve (AUC) values as the primary metric for classification accuracy (Naidu et al., 2023). To further confirm the robustness of the reported classification results, we computed 95% confidence intervals (CIs) for AUC estimates using DeLong variance estimates and performed DeLong’s test to compare different ROC curves (DeLong et al., 1988; Hajian-Tilaki et al., 1997). Additionally, we used bootstrap (resampling data with replacement) as an alternative method to estimate 95% CIs for AUC estimates, and when comparing AUC values between alternative models.

To quantify the contribution of each biological feature to model predictions, we applied Shapley Additive Explanations (SHAP) using the shap package (Lundberg and Lee, 2017). SHAP is based on cooperative game theory and computes feature attributions by considering all possible subsets of features, assigning each feature a Shapley value corresponding to its average marginal contribution to the model’s prediction. This framework provides a unified and model-agnostic measure of feature importance that is consistent across different model types. For each sample, SHAP values indicate both the magnitude and direction of a feature’s contribution to the predicted outcome. We aggregated SHAP values across samples to compute mean absolute SHAP values, providing a global measure of feature importance, and examined median SHAP values to assess the consistency of feature contributions across the dataset. This allowed us to distinguish features that consistently influenced predictions from those with sporadic or negligible impact. Importantly, SHAP analysis complements traditional feature importance measures by capturing both linear and non-linear effects, as well as interactions between features, within a unified interpretive framework (Ponce-Bobadilla et al., 2024).

## Results

### Dataset to define development of active disease in Mtb-exposed rhesus macaques

Previous study summarized data from multiple experiments in which rhesus macaques (RMs) were exposed to variable doses of different Mtb strains and followed over time with measurement of multiple blood components such as CRP and A/G ratio and frequency of major white blood cell subsets (**Figure 1A** and see Materials and methods for detail) (Gough et al., 2022). In total, we had data for 214 animals that is smaller than the fuller dataset of 225 animals presented previously (Gough et al., 2022); in part, our data do not include additional animals that had measurements of kynurenine, tryptophan, or their ratio that was added in Gough et al. (2022) for additional analyses. Every animal was classified as having active disease (“TB”, *n* = 134) or being asymptomatic (“no TB” or asymptomatic infection, *n* = 80) at the time of necropsy (**Figure 1A**). Out of all blood measurements, there were 20 primary markers (per animal) that were taken at the preinfection time point, at peak or bottom (nadir) values, or at necropsy (endpoint, **Supplementary Table S1**). Since the ratios of some blood cell subsets, e.g., neutrophil to lymphocyte or monocyte to lymphocyte, are thought to correlate with active disease in humans and animals (Yoon et al., 2013; La Manna et al., 2018; Berhane et al., 2019; Liu et al., 2022), we generated these ratios for all time points (see Materials and methods for detail). In total, this resulted in 31 features for further analyses (**Figure 1B**).

### Most measured markers do not show a clear separation between animals progressing to active disease vs. asymptomatic animals

First, we performed basic statistical analyses of the data. As expected, there was high variability in most of the measured features with CFU and CRP measurements having much higher standard deviations than the means (**Supplementary Table S1**). Means of over half of all features were statistically different between animals with TB vs. asymptomatic infection with CRP, A/G ratio, and lung CFU being the top based on the t value (**Supplementary Table S2**). Interestingly, nearly all metrics, based on ANOVA, were different at different times postinfection, suggesting that many of these features may be able to discriminate between symptomatic and asymptomatic animals (**Supplementary Table S3**). Analysis of correlations between individual features suggested that some of them vary between animals in a similar fashion, e.g., neutrophil or lymphocyte measurements at different times, which indicates that only few of such features will be important in the classification task (**Supplementary Figure S1**). PCA showed reasonable separation of animals with active disease vs. no disease (**Supplementary Figure S2**) with Lung.CFU, CRP.Endpoint, CRP.Peak, and A/G.Bottom and A/G.Endpoint being top features best explaining variability in the data (**Supplementary Table S4**).

We next inspected the distribution of all major features and their derivatives (i.e., ratios) for symptomatic and asymptomatic animals (**Figure 2** and **Supplementary Figure S3**). Interestingly, preinfection levels of any of the features did not differ between animals with TB or no TB (**Figure 2**i), both visually and statistically. This lack of separation was confirmed by low Kolmogorov–Smirnov (KS) statistics (*D* < 0.16, *p* > 0.05) and small 1-Wasserstein distances across features (*D_W_* < 1), indicating substantial overlap between the two cohorts at baseline. This suggests that these specific markers do not predetermine Mtb-exposed animals toward a particular disease trajectory (i.e., progression to disease or control/no disease).

Lung CFU exhibited the largest distributional separation between cohorts (**Figure 2a**i). For the no TB animals, the mean was 1.5 log_10_ CFU with a median of 1.25 log_10_ CFU, whereas for TB animals, the mean was 5.0 log_10_ CFU with a median of 4.88 log_10_ CFU (**Supplementary Table S1**). The separation between groups is further emphasized by the extreme values observed in each cohort, with the no TB group reaching approximately 4.7 log_10_ CFU compared to 7.1 log_10_ CFU in the TB group. This difference is supported statistically by a Cohen’s *d* of 3.09 and a KS statistic of 0.90, indicating strong separation between distributions. The magnitude of this separation is further reflected in a large 1-Wasserstein distance (*D_W_* = −2.59), capturing the substantial shift in distribution mass between the two cohorts.

CRP.Peak and CRP.Endpoint were also different between symptomatic and asymptomatic animals (**Figures 2**aii, iv). At peak levels, CRP exhibited a strong shift in distribution, supported by a KS statistic (*D* = 0.895, *p* < 0.001) and a large 1-Wasserstein distance (*D_W_* = 21.7), indicating minimal overlap and a pronounced right-tailed distribution in the TB cohort. Similarly, CRP levels at endpoint showed strong separation, with large KS statistic (*D* = 0.920, *p* < 0.001) and a large Wasserstein distance (*D_W_* = 19.2), further confirming the robustness of CRP as a discriminative feature. These metrics quantify the visual separation observed in the histograms and indicate that CRP captures both location and shape differences between the cohorts.

A/G ratio distributions were different for bottom and endpoint measurements (**Figure 2B**). In particular, A/G.Bottom showed strong separation driven by a left shift in the TB cohort relative to the no TB cohort. This was supported by a large magnitude Cohen’s *d* and a KS statistic (*D* = 0.786, *p* < 0.001), along with a somewhat small 1-Wasserstein distance (*D_W_* = 0.51), indicating both a shift in central tendency and differences in distribution geometry. These results suggest that A/G ratios capture systematic physiological differences between disease states.

Interestingly, monocytes did not show any meaningful differences at any time point examined, with low KS statistic (*D* < 0.25, *p* < 0.05 except of endpoint) and small Wasserstein distances across all comparisons, indicating substantial overlap between the cohorts. In contrast, both lymphocytes and neutrophils showed differences at endpoint measurements (**Figures 2C, D**). For example, Lymphocyte.Endpoint exhibited moderate separation, supported by a KS statistic (*D* = 0.557, *p* < 0.001) and a 1-Wasserstein distance *D_W_* = 14.8, indicating partial but not complete separation between classes. Similarly, neutrophil endpoint levels showed moderate separation, with significantly different KS statistics (*D* = 0.5, *p* < 0.001) and relatively large Wasserstein distances (*D_W_* = 15.8), reflecting differences in both mean and distribution shape.

Consistent with these observations, neutrophil-to-lymphocyte and monocyte-to-lymphocyte ratios showed differences at endpoint measurements but not at earlier timepoints, whereas monocyte-to-neutrophil ratios remained non-informative across all phases (**Supplementary Figure S3**). These results further support that discriminative signal is highly time-dependent and primarily emerges at later stages of disease progression, where both distributional shifts and changes in distribution geometry become more pronounced.

### Evaluation of individual features as predictors of TB

We next evaluated how individual features contribute to discriminating animals with active disease from asymptomatic animals using ML-based methods. We performed these analyses in three separate phases using different feature sets. First, we used all main features and their derivatives (i.e., ratios) to identify which measured variables were individually most informative for discriminating animals with active disease from animals without active disease. This analysis provides a direct estimate of the marginal predictive value of each feature when evaluated independently. Second, we repeated the same analysis after excluding all endpoint measurements, such as CRP.Endpoint and Lung.CFU. This analysis was designed to determine whether pre-endpoint features alone were sufficient to predict the development of active disease in RMs, rather than simply identifying disease status at necropsy. Third, we included only features measured at endpoint to determine which variables were most informative for diagnosing active disease at the final disease assessment.

We used three different ML-based methods to train models on selected features to discriminate between animals with active disease and asymptomatic animals: random forest (RF), logistic regression (LR), and linear support vector machine (L-SVM, **Figure 1B**). These models were chosen because they represent complementary classifier structures. RF is an ensemble tree-based model that can capture non-linear relationships and feature interactions without requiring strong distributional assumptions. In contrast, LR and L-SVM are linear classifiers, where classification is determined by a weighted combination of the input features. LR estimates class probabilities through a logistic function, whereas L-SVM identifies a separating hyperplane that maximizes the margin between classes. Thus, agreement between RF, LR, and L-SVM provides evidence that feature performance is not dependent on a single model architecture and that the observed class separation is likely driven by intrinsic structure in the dataset itself.

We used a standard 5-fold cross validation for model training and validation and generated multiple metrics characterizing model performance (see Materials and methods for detail). Because the primary goal of this section was to evaluate individual feature performance, we interpreted AUC values of ROC curves as a measure of how well each feature separated active disease from asymptomatic infection when used as the only predictor. Features with high AUC values across all three models were considered robust predictors, whereas features with inconsistent or low AUC values were interpreted as having limited marginal discriminatory value.

**Figure 3:**
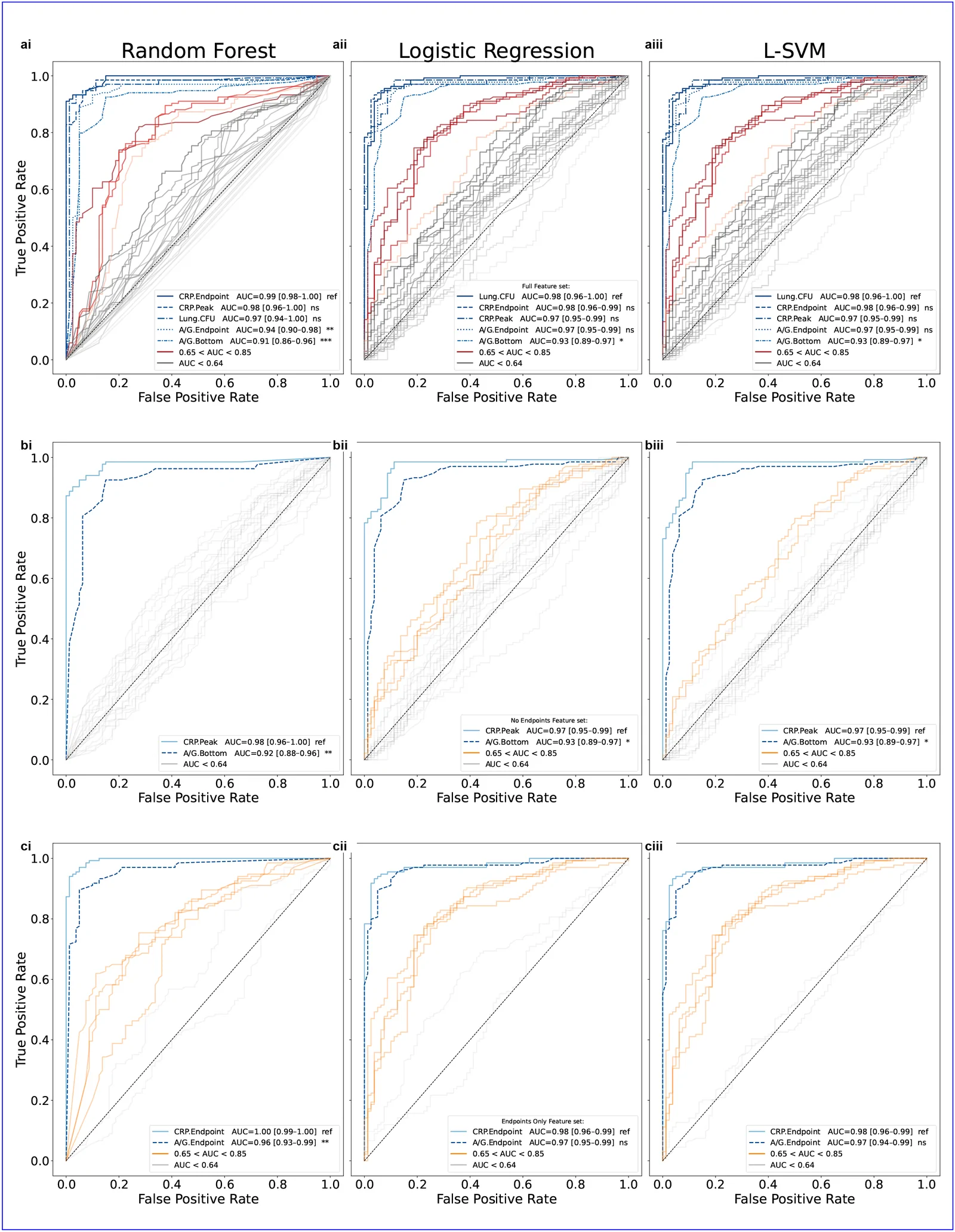
Blood-circulating proteins CRP and A/G ratio are best at discriminating between asymptomatic animals and animals with active disease in different settings. We performed single-feature evaluation (SFE) to quantify the predictive power of each feature independently for three models: Random Forest (RF, i), Logistic Regression (LR, ii), or Linear Support Vector Machine (L-SVM, iii). We show receiver operating characteristic (ROC) curves for each analysis with AUC values indicated for well/best-performing features. We performed our analysis using all features **(a)**, using all features except endpoint measurements **(b)**, or using only endpoint measurements **(c)**. Colors of the ROC curves indicate discriminatory ability of individual features (blue: AUC > 0.85, red/orange 0.65 ≤ AUC ≤ 0.85, and gray AUC< 0.65). We list 95% confidence intervals for each model and p values for comparing AUC from the top-performing model (denoted as “ref”) and other models using DeLong test. We used following notations: ns *p* > 0.05, *p ≤ 0.05, **p ≤ 0.01, ***p ≤ 0.001 (see also **Supplementary Tables S5**-**S9**).

In the analysis where only a single feature was selected for model training while considering all available features, Lung.CFU was the most influential feature discriminating between TB vs. no TB in L-SVM and LR models (AUC = 0.99), whereas in the RF model, CRP.Endpoint was the best predictor (**Figure 3A**). Interestingly, independently of the ML method, five features (Lung.CFU, CRP.Peak, CRP.Endpoint, A/G.Bottom, and A/G.Endpoint) were superior to all other features at discriminating between active disease vs. no disease, with AUC > 0.90 (**Figure 3A**). We additionally computed 95% confidence intervals (CIs) for the AUC estimates and performed DeLong’s test comparing each feature-specific AUC against AUC of the top performing feature within each model (**Supplementary Table S5**). Interestingly, statistically only top four features were not different from the top-performing feature (**Figure 3a**), suggesting that A/G.Bottom even with a high AUC value was still a slightly worse predictor of active disease as compared to top-performing features (CRP.Endpoint or Lung.CFU). The consistency of top five features across RF, LR, and L-SVM models indicates that their predictive value is not model-specific. Rather, these features appear to provide strong marginal separation between disease classes, such that both linear and non-linear classifiers display similar discriminatory performance.

In contrast, even the best-performing remaining features, such as M-L.Endpoint or Lymphocytes.Endpoint, had maximum AUC ≤0.80, which was substantially lower than the AUC values observed for the top-performing features and was statistically different from the top feature (**Figure 3a**; **Supplementary Table S5**). This performance gap was further supported by the 95% CIs for AUC. Most of the 31 features had very low predictive power, with some even reaching AUC< 0.5, lower than that expected from a random coin-toss classifier. These low-AUC features likely contain little independent discriminatory signal when evaluated individually, or their relationship to disease status may depend on multivariate context not captured by single-feature models.

This ranking of features was further supported by SHAP analysis (**Supplementary Figures S4**–**S6**). SHAP values quantify the contribution of each feature to model predictions, allowing us to determine whether a feature with a high AUC also had a strong influence on model decision-making. For Lung.CFU, the mean absolute SHAP value was 0.139 with an absolute median of 0.129 for L-SVM; for LR, the absolute mean was 0.163 with an absolute median of 0.160; and for RF, the absolute mean was 0.074 with an absolute median of 0.067. The close agreement between mean and median SHAP values across all three models indicates that Lung.CFU contributed consistently across samples rather than being driven by a small number of influential outliers. This supports the interpretation that Lung.CFU is a stable and dominant endpoint predictor of disease status.

Similarly, CRP.Endpoint and A/G.Endpoint showed strong model contribution across all three classifiers (e.g., for RF, we found SHAP mean and median of 0.089 and 0.0801 for CRP and 0.051 and 0.047 for A/G, respectively; **Supplementary Figure S4**). CRP.Endpoint was also one of the strongest single-feature predictors, ranking first in RF with an AUC of 0.99 and second in LR and L-SVM with an AUC value of approximately 0.98. Although SHAP values for A/G.Endpoint were lower than those observed for Lung.CFU and CRP.Endpoint, their consistency across models indicates that A/G.Endpoint provided reproducible discriminatory information. This result is consistent with the single-feature ROC analysis, where A/G.Endpoint achieved high AUC values across RF, LR, and L-SVM.

In contrast, Lymphocyte.Endpoint had substantially weaker SHAP contributions. For RF, the mean SHAP value was 9.84 × 10^−3^ with a median of 9.79 × 10^−3^ for LR, the mean SHAP value was 2.56 × 10^−3^ with a median of 0.0; and for L-SVM, the mean SHAP value was 4.89 × 10^−3^ with a median of 0.0. These low values support the interpretation that Lymphocyte.Endpoint had limited influence on model predictions, despite achieving moderate AUC values in some models. The near-zero median SHAP values for LR and L-SVM models suggest that, for many samples, this feature did not materially affect the classification decision. We reached similar conclusions for N/L or M/L ratios (**Supplementary Figure S4**).

Calibration analyses, resampling-based feature evaluation and Youden’s J statistic further supported Lung.CFU and CRP values as top predictors discriminating between asymptomatic animals and animals with active disease (**Supplementary Tables S6**, **S7**; **Supplementary Figures S7**, **S8**). Youden’s J statistic summarizes the performance of a binary classifier at a selected threshold by combining sensitivity and specificity, where *J* = sensitivity + specificity − 1. Thus, a high Youden’s J value indicates that a feature or model threshold achieves both high true-positive detection and high true-negative discrimination. The agreement between AUC rankings, SHAP values, and Youden’s J therefore strengthens the conclusion that Lung.CFU and CRP-related features are not only globally discriminative but also useful at specific decision thresholds relevant for classification (**Supplementary Table S7**).

Restricting the analysis to features that exclude endpoint measurements resulted in only two features, CRP.Peak and A/G.Bottom, that accurately discriminated between animals with active disease and asymptomatic animals (**Figure 3b**). But even then, A/G.Bottom was a statistically worse predictor than CRP.Peak based on DeLong’s test, and none of the other non-endpoint features were predictive at reasonable levels (**Supplementary Table S8**). This suggests that, before endpoint, the strongest signal predicting development of active disease is concentrated in one or two blood protein markers rather than broadly distributed across all measured variables.

Finally, when only endpoint features were included, while excluding Lung.CFU because CFU measurements are more difficult to obtain than blood-based markers, CRP.Endpoint and A/G.Endpoint again emerged as the best predictors at diagnosing the disease. AUC for A/G.Endpoint was statistically smaller than that of top-performing feature (CRP.Endpoint) for the RF-based model but not for other two models (**Figure 3c**). M/L and N/L ratios, as well as lymphocytes, showed poorer accuracy (**Figure 3c**; **Supplementary Table S9**). These results indicate that blood-based markers, particularly CRP and A/G ratios, retain strong discriminatory ability to diagnose TB in rhesus macaques even when bacterial burden measurements are excluded. This finding is important because it suggests that experimentally accessible blood-based measurements may approximate disease classification performance otherwise dominated by Lung.CFU.

### Few features are sufficient to discriminate between active disease or no disease in different conditions

**Figure 4:**
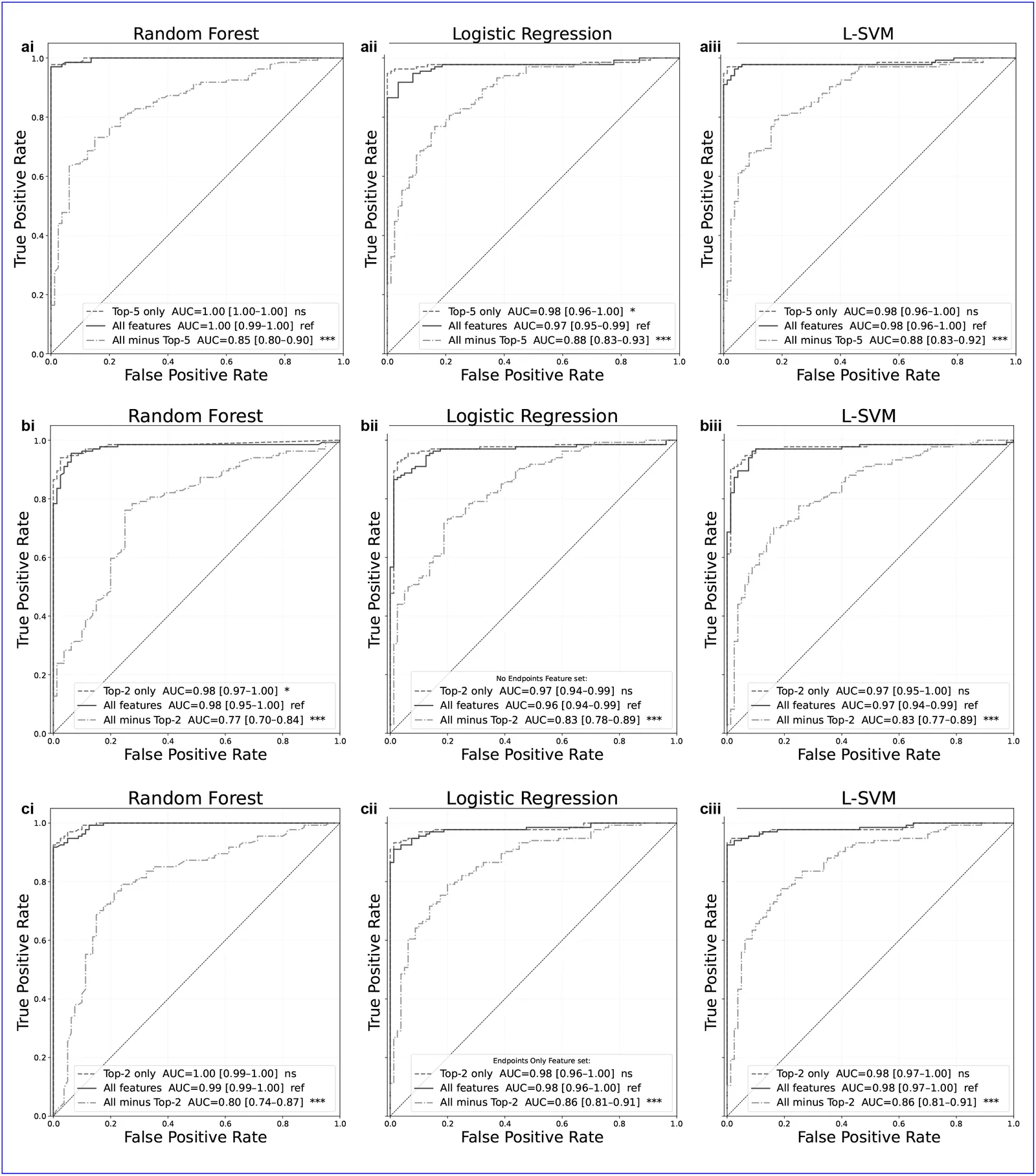
Few top features are sufficient to robustly discriminate between asymptomatic animals or animals with active disease. Similarly to **Figure 3**, we used RF (i), LR (ii), or L-SVM (iii) to train models to discriminate between asymptomatic animals or animals with TB. We performed our analysis using either all features, top 5 (most predictive) features, or all features without top 5 predictive features [**(a)**, see **Supplementary Table S1**)], using all features except endpoint measurements, only top two features excluding endpoints, or all features without top two features excluding endpoints **(b)**, or using only all endpoint measurements, top two endpoint measurements, or all endpoints without top two **(c)**. Other notations are the same as in **Figure 3**.

We next explored whether subsets of the best-performing features would perform comparably to all features for a given classification task (**Figure 4**). Similar to the previous results (e.g., **Figure 3**), the outcome of these analyses was approximately independent of the ML method used to train models on the data. In particular, the model trained using only the top 5 features had predictive performance similar to the model using all 31 features (AUC ≥ 0.97, **Figure 4a**); this was also supported statistically by 95% CIs and from DeLong’s test (**Supplementary Table S5**). Importantly, excluding the top 5 features resulted in a marked reduction in model prediction quality (AUC ≤ 0.88, **Figure 4a**), indicating that most of the discriminatory signal was concentrated in this small subset of features.

Similarly, when excluding endpoint measurements, CRP.Peak and A/G.Bottom together were as predictive of active disease as all features combined (AUC ≥ 0.96, **Figure 4b**), whereas excluding these two top features reduced the ability of the model to discriminate between asymptomatic animals and those with active disease (AUC ≤ 0.83). We found similar conclusions when only including endpoint measurements (**Figure 4c**). Taken together, these results suggest that blood-based proteins such as CRP and A/G ratio have higher predictive power for diagnosing active disease or predicting progression to active disease in Mtb-exposed RMs than other proposed markers such as N/L ratio. Using the LR model as an example, we identified values of CRP.Peak of 5.2 µg/mL and A/G.Bottom of 0.88 as individual thresholds associated with active disease vs. asymptomatic status. In the LR model where both features are used to define active disease (**Figure 4**bii), A/G.Bottom ≤ 1 and variable levels of CRP were predictive of active disease (**Supplementary Figure S9**).

## Discussion

The aim of our study was to determine the features that predict development of or allow to diagnose active disease in Mtb-exposed RMs. By using standard statistical tools and more advanced ML-based analyses, we found that of 31 features contained in our data, the most significant features were Lung CFU (measured at necropsy/endpoint), CRP levels (measured either as peak or at endpoint), and A/G ratio levels (measured as bottom or at endpoint, **Figure 3**). Other features such as lymphocyte concentration and N/L or M/L ratio had relatively low predictive power in any of the analyses (**Supplementary Table S5**). Interestingly, while the type of ML method used for analysis often influences accuracy of the trained model, it was not the case for our data where the results were relatively agnostic to the ML model choice (e.g., **Supplementary Table S5**). Importantly, we observed that model performance remained stable under substantial reductions in feature dimensionality where selection of only two or five features was sufficient for ML-based models to achieve the same accuracy as the models trained using the full-feature set (**Figure 4**). In that analysis, we also found that excluding the top features from the all features set dramatically reduces the model accuracy suggesting that the state of active disease in Mtb-exposed RMs is determined by one or two features. From a modeling perspective, it indicates that the classification task is effectively low-dimensional, where a small number of dominant features capture the majority of the separability between classes, whereas additional features contribute diminishing or redundant information.

High lung CFU levels are a well-established marker in TB, and elevated CRP levels are an expected consequence of immune response to infection and/or disseminated nature of Mtb infection in RMs. We were therefore not surprised to observe that these features dominated in defining accuracy of the trained models. However, the magnitude of their contribution relative to other available features was notable (**Supplementary Figure S4**). For example, per SHAP analysis for Lung.CFU in the L-SVM model, the mean absolute SHAP value was 0.139 with a median of 0.129, compared to Lymphocyte.Endpoint, where the L-SVM mean SHAP value was 4.89 × 10^−3^ with a median of 0.0 (**Supplementary Figure S4**). This indicates that not only was the model able to effectively utilize Lung.CFU as a classification feature, but that this feature induces a strong and consistent separation between classes across samples. In contrast, the near-zero median SHAP value for Lymphocyte.Endpoint suggests that this feature contributes minimally in most cases, explaining its lower importance ranking. From a structural standpoint, this reinforces that model predictions are driven primarily by a small subset of dominant features, rather than distributed contributions across many variables. A/G ratio levels across all three models were consistently among the top 5 features at peak and necropsy timepoints although statistically the AUC value for the A/G ratio was slightly lower as compared to the top-performing feature. From our analysis, we propose that a CRP increase above 5.2 µg/ml or an A/G ratio drop below 0.88 are individually highly predictive of active disease in Mtb-exposed RMs. In case when measurements of both features are available, our ML-based analysis suggests a combination of CRP and A/G ratio values defining active disease (**Supplementary Figure S9**). These values could be used to assist in diagnosing active disease in Mtb-exposed rhesus macaques.

In our analysis, white blood cell-based markers such as N/L or M/L ratio did not play a major role at predicting TB development or at diagnosing active disease (**Supplementary Table S5**). Neutrophils and lymphocytes acted as secondary markers relative to the dominant features (AUC< 0.8), whereas monocytes played little to no role in the differentiation task. This is notable given the emphasis in the literature on the importance of neutrophils, lymphocytes, and their derivatives in disease progression, suggesting that the neutrophil-to-lymphocyte ratio can outperform CRP levels in diagnostic settings (Yoon et al., 2013; La Manna et al., 2018; Berhane et al., 2019; Liu et al., 2022). This may indicate differences in TB development between RMs and humans—Mtb-exposed RMs typically develop primary TB whereas in humans, post-primary TB is more common (Cohen et al., 2022). Other factors such as differences in study design, cohort composition, or the distinction between diagnostic-focused versus progression-focused analyses may also be important (but see also limitations of our study).

Our main conclusion that CRP and A/G ratio are best correlates of active disease in Mtb-exposed RMs is not entirely surprising. For example, WHO recommends using CRP levels as an additional metric when diagnosing TB in humans with the caveat that elevated CRP levels are not exclusive to TB (WHO, 2021). Increased levels of CRP, an acute phase protein produced in the liver, is a typical indicator of ongoing immune response; in TB, higher CRP values may be indicative of Mtb dissemination to extrapulmonary sites—such dissemination is typical in highly susceptible RM model in setting of primary Mtb infection (Lin et al., 2014; Moule and Cirillo, 2020). The A/G ratio may change in Mtb infection because of both declining levels of albumin and increasing levels of globulin. Albumin levels may decline because of systemic inflammation during Mtb infection, and globulin levels rise in part to alpha-globulin production and antibody response to Mtb (Don and Kaysen, 2004; Jacobs et al., 2016). In Mtb-infected RMs, the A/G ratio seems to primarily change because of increase in globulin levels (unpublished observations). CRP and globulin levels have shown moderate precision at diagnosing TB or predicting success of TB treatment in patients (Bapat et al., 2015; Shingdang et al., 2016; Yoon et al., 2017; Gaeddert et al., 2024). Our results broadly align with the previous findings of Gough et al. (2022). We were able to quantitatively identify significant features from the dataset due to strong signal, and similarly observed qualitative relationships between elevated CRP levels, increased neutrophil levels, decreased A/G ratios (suggesting higher globulin levels), and reduced lymphocyte levels in RMs with TB.

Our study has several limitations. While our dataset is substantial with over 200 animals, it is still relatively small (e.g., as compared to some human cohort studies). The absence of a continuous time-course dataset required the assumption that time phases (e.g., peak, bottom) are aligned across features that may not be the case. While we do not believe this assumption invalidates our primary conclusions, the presence of inverse correlations in subsets of features (**Supplementary Figure S1**) suggests that proper temporal alignment may yield more precise characterization of these relationships. Lack of temporal data led to some uncertainty of calculated ratios of different cell subsets that may also explain their relatively poor predictive power as compared to blood-located proteins. Furthermore, given the structure of the dataset, we were limited to relatively simple model classes that had at most only 31 features. While regression methods, decision trees, and kernel-based approaches were appropriate for the given feature space, these models do not explicitly capture temporal dynamics or higher-order interactions. The observed stability of model performance across feature subsets suggests that the classification problem is dominated by strong marginal effects; however, with the introduction of temporal information and larger datasets, these models may overgeneralize or fail to capture more complex dynamics. In such cases, more expressive approaches, such as gradient boosted trees, non-linear support vector machines, or generalized additive models may be required.

Our work opens avenue for future research. The primary direction for extending this study is the incorporation of a fully time-resolved dataset, enabling direct analysis of disease progression dynamics. A time-specific dataset would allow for the identification of temporal relationships and potentially reveal predictive signals that are obscured in discretized timepoints. Additionally, such data would enable the use of a broader class of models capable of capturing temporal dependencies, including MultiLayer Perceptrons (MLPs), recurrent architectures, and survival-based models such as Cox proportional hazards models. With these models, it would be possible to study the kinetics of immune response, providing deeper insight into the mechanisms underlying disease progression. Adding other data modalities, for example, periodic animal scans using positron emission tomography–computed tomography (PET/CT) may add additional information on the location of lung lesions and/or sites of inflammation (Hurtado et al., 2026) and thus may help further define features of progression of Mtb-exposed animals to active disease. In our initial exploratory analysis, we observed that certain features exhibited strong correlations with each other while contributing minimally to model performance. A more comprehensive dataset would allow for deeper investigation of these relationships, particularly in a multivariate setting where interactions between features may reveal previously hidden signals. Increasing the number of samples and timepoints would further enhance the ability to characterize these mechanisms, ultimately contributing to a more complete understanding of factors defining progression of Mtb-exposed monkeys to TB.

## Data Availability

Publicly available datasets were analyzed in this study. This data can be found here: https://github.com/dahalfblood/tb-ml. Analyses have primarily been performed in python (version 3.14); scripts for data analysis and machine learning are provided on GitHub: https://github.com/dahalfblood/tb-ml.
