# Supplementary material for: Machine learning-based feature selection suggests circulating proteins as best at predicting and diagnosing tuberculosis in Mycobacterium tuberculosis-exposed rhesus macaques
Source: Front Cell Infect Microbiol. 2026 Aug 6;16:1880496. doi: 10.3389/fcimb.2026.1880496 (PMC13489891; doi:10.3389/fcimb.2026.1880496)
Supplement: Supplementary file 1 [file DataSheet1.pdf]

Machine learning-based feature selection suggests circulating proteins as best at predicting and diagnosing tuberculosis in Mycobacterium tuberculosis-exposed rhesus macaques

Taylor A. L. Woodard, Smriti Mehra, Deepak Kaushal, Vitaly V. Ganusov

Supplemental Information

	Max	Mean	Median	Min	Mode	Std Dev
Lung.CFU	50366.47 / 12500000.0	1074.98 / 1022868.13	18.75 / 75000.0	0.6 / 285.23	1.0 / 33333.0	5720.05 / 2444955.18
A/G.Pre-infection	2.3 / 2.3	1.54 / 1.55	1.5 / 1.6	1.1 / 0.6	1.7 / 1.6	0.25 / 0.26
A/G.Peak	3.1 / 2.3	1.84 / 1.67	1.8 / 1.7	1.1 / 0.8	1.7 / 1.7	0.43 / 0.26
A/G.Bottom	1.9 / 1.4	1.13 / 0.62	1.1 / 0.6	0.2 / 0.3	1.1 / 0.6	0.28 / 0.19
A/G.Endpoint	2.0 / 1.6	1.4 / 0.64	1.4 / 0.6	0.7 / 0.3	1.3 / 0.5	0.33 / 0.21
Lymphocytes.Pre-infection	72.8 / 78.0	38.65 / 40.65	36.6 / 40.2	13.5 / 13.8	31.3 / 30.3	14.39 / 12.56
Lymphocytes.Peak	77.5 / 78.0	47.88 / 45.33	47.7 / 45.3	20.5 / 18.9	31.4 / 39.2	13.12 / 11.53
Lymphocytes.Bottom	54.0 / 39.1	24.11 / 17.28	22.4 / 16.2	7.2 / 3.4	17.5 / 11.0	9.87 / 6.53
Lymphocytes.Endpoint	66.8 / 47.3	34.46 / 19.66	33.0 / 18.3	8.3 / 5.0	17.2 / 13.0	12.9 / 8.26
Monocytes.Pre-infection	10.2 / 9.4	4.2 / 4.4	4.0 / 4.1	1.9 / 1.5	4.2 / 3.0	1.57 / 1.59
Monocytes.Peak	26.1 / 22.0	6.97 / 7.73	6.4 / 7.4	2.2 / 3.1	4.2 / 7.7	3.39 / 2.88
Monocytes.Bottom	7.0 / 7.6	3.12 / 3.47	3.0 / 3.4	1.0 / 0.1	2.5 / 3.7	1.13 / 1.4
Monocytes.Endpoint	11.2 / 22.0	4.99 / 6.21	4.7 / 5.7	1.8 / 0.1	3.6 / 5.7	2.01 / 3.03
Neutrophils.Pre-infection	82.9 / 80.5	53.55 / 51.33	54.5 / 51.5	16.7 / 14.7	60.9 / 34.3	14.61 / 12.94
Neutrophils.Peak	89.9 / 93.7	68.49 / 75.68	71.3 / 76.2	37.1 / 50.7	59.2 / 63.6	11.42 / 8.51
Neutrophils.Bottom	72.2 / 76.2	42.79 / 46.14	43.6 / 47.2	16.1 / 14.7	27.8 / 59.0	14.13 / 12.24
Neutrophils.Endpoint	88.5 / 91.0	56.29 / 72.04	56.2 / 72.5	23.6 / 40.3	47.7 / 64.2	14.16 / 10.14
CRP.Pre-infection	0.0 / 0.0	0.0 / 0.0	0.0 / 0.0	0.0 / 0.0	0.0 / 0.0	0.0 / 0.0
CRP.Peak	8.47 / 96.9	1.29 / 22.98	0.57 / 17.44	0.0 / 0.0	0.0 / 6.27	2.11 / 17.24
CRP.Endpoint	3.6 / 84.0	0.24 / 19.41	0.0 / 13.85	0.0 / 0.05	0.0 / 0.05	0.71 / 16.35

Supplemental Table S1: Summary statistics for all features for asymptomatic animals or animals with TB. We show the maximum, mean, median, minimum, mode, and standard deviation for each feature in the dataset. The values are presented as for asymptomatic/symptomatic animals. The features are grouped by category, with the first column showing the feature name.

Feature	t value	p value	FDR
CRP.Endpoint	-26.634	<0.001	<0.001
CRP.Peak	-22.603	<0.001	<0.001
Lung.CFU	-19.980	<0.001	<0.001
A/G.Endpoint	18.064	<0.001	<0.001
A/G.Bottom	14.219	<0.001	<0.001
Lymphocytes.Endpoint	9.360	<0.001	<0.001
L-M.Endpoint	8.990	<0.001	<0.001
Neutrophils.Endpoint	-8.661	<0.001	<0.001
L-N.Endpoint	8.486	<0.001	<0.001
Lymphocytes.Bottom	5.600	<0.001	<0.001
Neutrophils.Peak	-4.935	<0.001	<0.001
L-N.Bottom	4.406	<0.001	<0.001
L-M.Bottom	4.150	<0.001	<0.001
A/G.Peak	3.270	0.001	0.003
Monocytes.Endpoint	-3.169	0.002	0.004
L-N.Peak	3.085	0.002	0.005
L-M.Peak	2.708	0.008	0.014
Monocytes.Peak	-2.015	0.046	0.079
Neutrophils.Bottom	-1.783	0.077	0.125
Monocytes.Bottom	-1.746	0.082	0.128
Lymphocytes.Peak	1.433	0.154	0.227
Lymphocytes.Pre-infection	-1.176	0.241	0.340
M-N.Pre-infection	-1.136	0.257	0.347
M-N.Endpoint	1.081	0.281	0.363
Neutrophils.Pre-infection	1.016	0.311	0.386
Monocytes.Pre-infection	-0.966	0.336	0.400
L-N.Pre-infection	-0.374	0.709	0.814
M-N.Bottom	0.245	0.807	0.893
L-M.Pre-infection	0.150	0.881	0.920
A/G.Pre-infection	-0.130	0.897	0.920
M-N.Peak	-0.100	0.920	0.920

Supplemental Table S2: List of features best differing between asymptomatic animals and animals with active disease. We performed t-test for each feature for asymptomatic animals and animals with TB. We list t value from the test, corresponding p value from a test of no difference, and false discovery rate-corrected p value.

Base Feature	# of measurements	F Value	p Value
Neutrophils	4	2878.763	0.000
Monocytes	4	1386.316	0.000
Lymphocytes	4	1372.601	0.000
L-M	4	869.476	0.000
A/G	4	862.503	0.000
M-N	4	405.294	0.000
L-N	4	344.630	0.000
CRP	2	5.659	0.018

Supplemental Table S3: Nearly all features change over time since infection. We used ANOVA to quantify differences in feature values at different time points (pre-infection, peak, bottom, or endpoint). The table lists each base feature along with its corresponding phases, number of measurements, F-statistic, and p-value. Note that CRP values were only available for the peak and endpoint (pre-infection levels were 0 for all animals).

Feature	PC1	PC2	$\|\cdot\|$
Lung.CFU	-0.1850	0.0980	0.2094
A/G.Bottom	0.2207	0.2970	0.3700
A/G.Endpoint	0.2284	0.3171	0.3909
CRP.Endpoint	-0.2348	-0.2572	0.3482
CRP.Peak	-0.2379	-0.2587	0.3514
Neutrophils.Endpoint	-0.3478	-0.0212	0.3484
Lymphocytes.Endpoint	0.3543	0.0704	0.3612

Supplemental Table S4: PCA loadings for the top features contributing to the first two principal components on the full feature set. We performed PCA analysis for all 20 features; we show the coefficients of each feature on PC1 and PC2, as well as the Euclidean norm ($\|\cdot\|$) summarizing their overall contribution in the 2D projection.

Rank	RF Feature	RF AUC	LR Feature	LR AUC	SVM Feature	SVM AUC
1	CRP.Endpoint	0.992	Lung.CFU	0.980	Lung.CFU	0.979
2	CRP.Peak	0.976	CRP.Endpoint	0.976	CRP.Endpoint	0.976
3	Lung.CFU	0.968	CRP.Peak	0.974	CRP.Peak	0.974
4	A/G.Endpoint	0.938	A/G.Endpoint	0.968	A/G.Endpoint	0.967
5	A/G.Bottom	0.910	A/G.Bottom	0.928	A/G.Bottom	0.929
6	L-M.Endpoint	0.800	L-M.Endpoint	0.834	L-M.Endpoint	0.836
7	Lymphocytes.Endpoint	0.781	Lymphocytes.Endpoint	0.828	Lymphocytes.Endpoint	0.830
8	L-N.Endpoint	0.779	L-N.Endpoint	0.823	L-N.Endpoint	0.825
9	Neutrophils.Endpoint	0.739	Neutrophils.Endpoint	0.806	Neutrophils.Endpoint	0.807
10	L-M.Bottom	0.659	L-M.Bottom	0.708	L-M.Bottom	0.708
11	Neutrophils.Peak	0.613	Lymphocytes.Bottom	0.690	Lymphocytes.Bottom	0.692
12	Lymphocytes.Bottom	0.611	Neutrophils.Peak	0.673	Neutrophils.Peak	0.673
13	Monocytes.Endpoint	0.591	L-N.Bottom	0.651	L-N.Bottom	0.651
14	L-M.Peak	0.579	Monocytes.Endpoint	0.624	Monocytes.Endpoint	0.622
15	L-N.Bottom	0.568	A/G.Peak	0.614	A/G.Peak	0.613
16	A/G.Peak	0.568	L-N.Peak	0.603	L-N.Peak	0.603
17	L-N.Peak	0.565	L-M.Peak	0.602	L-M.Peak	0.602
18	Monocytes.Peak	0.549	Monocytes.Peak	0.591	Monocytes.Peak	0.592
19	Monocytes.Bottom	0.529	Monocytes.Bottom	0.569	Monocytes.Bottom	0.565
20	Lymphocytes.Pre-infection	0.528	Neutrophils.Bottom	0.563	Neutrophils.Bottom	0.561
21	Neutrophils.Bottom	0.525	Lymphocytes.Peak	0.547	Lymphocytes.Peak	0.543
22	L-N.Pre-infection	0.517	Lymphocytes.Pre-infection	0.540	Lymphocytes.Pre-infection	0.540
23	A/G.Pre-infection	0.497	Neutrophils.Pre-infection	0.528	Neutrophils.Pre-infection	0.533
24	Neutrophils.Pre-infection	0.492	M-N.Pre-infection	0.518	M-N.Pre-infection	0.518
25	M-N.Pre-infection	0.492	Monocytes.Pre-infection	0.510	M-N.Endpoint	0.508
26	Lymphocytes.Peak	0.489	M-N.Endpoint	0.500	Monocytes.Pre-infection	0.505
27	M-N.Endpoint	0.476	L-N.Pre-infection	0.482	L-M.Pre-infection	0.500
28	M-N.Peak	0.468	A/G.Pre-infection	0.463	M-N.Peak	0.495
29	L-M.Pre-infection	0.454	M-N.Peak	0.456	L-N.Pre-infection	0.489
30	Monocytes.Pre-infection	0.448	L-M.Pre-infection	0.432	A/G.Pre-infection	0.458
31	M-N.Bottom	0.428	M-N.Bottom	0.395	M-N.Bottom	0.395

Supplemental Table S5: Top features best discriminating between asymptomatic monkeys and monkeys with TB are consistent between different ML methods. We used random forest (RF), logistic regression (LR), or linear support vector machine (SMV) methods and a single feature to discriminate between asymptomatic animals and animals with active disease. We show AUC for individual features sorted from highest to lower values (see also **Figure 3**).

Feature	$RF_{\Delta AUC}$	$LR_{\Delta AUC}$	$SVM_{\Delta AUC}$	$Mean_{\Delta AUC}$	Avg Rank
Lung.CFU	0.0142	0.0912	0.0567	0.0541	1.0000
CRP.Endpoint	0.0108	0.0080	0.0179	0.0122	3.0000
CRP.Peak	0.0022	0.0110	0.0216	0.0116	2.6667
A/G.Endpoint	0.0010	0.0144	0.0147	0.0100	3.3333
A/G.Bottom	-0.0001	0.0023	0.0023	0.0015	8.0000
Lymphocytes.Endpoint	-0.0001	0.0014	0.0010	0.0007	8.6667
Lymphocytes.Peak	-0.0002	0.0019	0.0005	0.0007	9.3333
Monocytes.Peak	-0.0000	0.0011	0.0001	0.0004	8.1667
Neutrophils.Bottom	0.0000	0.0005	0.0002	0.0002	8.8333
Neutrophils.Endpoint	-0.0000	0.0008	-0.0003	0.0002	10.3333

Supplemental Table S6: Lung bacterial burden and CRP emerged as the most predictive features. We performed permutation importance analysis for each of our machine learning models. We computed the change in AUC (ΔAUC) after permuting each feature, averaged over 100 permutations. The table shows the ΔAUC for each feature and model, as well as the mean ΔAUC across models. The threshold ($\overline{\Delta AUC} \geq 0.002$) emphasises the most stable features across the rankings.

Feature	AUC	True Positive	False Positive	Threshold	J-Score
CRP.Endpoint	0.99	0.92	0.00	0.89	0.92
Lung.CFU	0.97	0.93	0.04	0.75	0.90
CRP.Peak	0.96	0.95	0.06	0.91	0.89
A/G.Endpoint	0.93	0.89	0.05	0.92	0.84
A/G.Bottom	0.91	0.93	0.15	0.34	0.78
Lymphocytes.Endpoint	0.77	0.75	0.23	0.76	0.53
Neutrophils.Endpoint	0.73	0.76	0.30	0.75	0.46
Neutrophils.Peak	0.63	0.84	0.61	0.61	0.22
Lymphocytes.Bottom	0.63	0.77	0.51	0.65	0.26
Monocytes.Endpoint	0.61	0.54	0.31	0.65	0.23
A/G.Peak	0.58	0.84	0.65	0.66	0.19
Neutrophils.Bottom	0.55	0.53	0.40	0.67	0.13
Monocytes.Bottom	0.55	0.32	0.19	0.67	0.13
Monocytes.Peak	0.55	0.77	0.61	0.63	0.16
Lymphocytes.Pre-infection	0.54	0.66	0.51	0.64	0.14
Monocytes.Pre-infection	0.53	0.49	0.38	0.63	0.12
Lymphocytes.Peak	0.50	0.92	0.82	0.59	0.09
Neutrophils.Pre-infection	0.50	0.82	0.72	0.60	0.10
A/G.Pre-infection	0.46	0.84	0.84	0.59	0.01

Supplemental Table S7: Youden's J Statistic shows a clear separation between the most important features and the features that are potentially contributing to noise and overfitting. Per-feature ROC curves were computed using 5-fold CV. The table shows model statistics for each feature, including the Area Under the Curve (AUC), True Positive Rate (TPR), False Positive Rate (FPR), Threshold, and Youden's J statistic. The features are ordered by AUC, with the highest AUC features at the top. The J-Score is calculated as TPR - FPR, and is used to determine the optimal threshold for each feature. The features with the highest J-Score are considered the most important for the classification task.

Rank	RF Feature	RF AUC	LR Feature	LR AUC	SVM Feature	SVM AUC
1	CRP.Peak	0.976	CRP.Peak	0.974	CRP.Peak	0.974
2	A/G.Bottom	0.910	A/G.Bottom	0.928	A/G.Bottom	0.929
3	L-M.Bottom	0.659	L-M.Bottom	0.708	L-M.Bottom	0.708
4	Neutrophils.Peak	0.613	Lymphocytes.Bottom	0.690	Lymphocytes.Bottom	0.692
5	Lymphocytes.Bottom	0.611	Neutrophils.Peak	0.673	Neutrophils.Peak	0.673
6	L-M.Peak	0.579	L-N.Bottom	0.651	L-N.Bottom	0.651
7	L-N.Bottom	0.568	A/G.Peak	0.614	A/G.Peak	0.613
8	A/G.Peak	0.568	L-N.Peak	0.603	L-N.Peak	0.603
9	L-N.Peak	0.565	L-M.Peak	0.602	L-M.Peak	0.602
10	Monocytes.Peak	0.549	Monocytes.Peak	0.591	Monocytes.Peak	0.592
11	Monocytes.Bottom	0.529	Monocytes.Bottom	0.569	Monocytes.Bottom	0.565
12	Lymphocytes.Pre-infection	0.528	Neutrophils.Bottom	0.563	Neutrophils.Bottom	0.561
13	Neutrophils.Bottom	0.525	Lymphocytes.Peak	0.547	Lymphocytes.Peak	0.543
14	L-N.Pre-infection	0.517	Lymphocytes.Pre-infection	0.540	Lymphocytes.Pre-infection	0.540
15	A/G.Pre-infection	0.497	Neutrophils.Pre-infection	0.528	Neutrophils.Pre-infection	0.533
16	Neutrophils.Pre-infection	0.492	M-N.Pre-infection	0.518	M-N.Pre-infection	0.518
17	M-N.Pre-infection	0.492	Monocytes.Pre-infection	0.510	Monocytes.Pre-infection	0.505
18	Lymphocytes.Peak	0.489	L-N.Pre-infection	0.482	L-M.Pre-infection	0.500
19	M-N.Peak	0.468	A/G.Pre-infection	0.463	M-N.Peak	0.495
20	L-M.Pre-infection	0.454	M-N.Peak	0.456	L-N.Pre-infection	0.489
21	Monocytes.Pre-infection	0.448	L-M.Pre-infection	0.432	A/G.Pre-infection	0.458
22	M-N.Bottom	0.428	M-N.Bottom	0.395	M-N.Bottom	0.395

Supplemental Table S8: Only two features have a high predictive power in discriminating between asymptomatic animals and animals with TB when taking into account all measurements prior to endpoint. We performed similar analyses as in **Supplemental Table S5** except we excluded endpoint features from the analysis.

Rank	RF Feature	RF AUC	LR Feature	LR AUC	SVM Feature	SVM AUC
1	CRP.Endpoint	0.992	CRP.Endpoint	0.976	CRP.Endpoint	0.976
2	A/G.Endpoint	0.938	A/G.Endpoint	0.968	A/G.Endpoint	0.967
3	L-M.Endpoint	0.800	L-M.Endpoint	0.834	L-M.Endpoint	0.836
4	Lymphocytes.Endpoint	0.781	Lymphocytes.Endpoint	0.828	Lymphocytes.Endpoint	0.830
5	L-N.Endpoint	0.779	L-N.Endpoint	0.823	L-N.Endpoint	0.825
6	Neutrophils.Endpoint	0.739	Neutrophils.Endpoint	0.806	Neutrophils.Endpoint	0.807
7	Monocytes.Endpoint	0.591	Monocytes.Endpoint	0.624	Monocytes.Endpoint	0.622
8	M-N.Endpoint	0.476	M-N.Endpoint	0.500	M-N.Endpoint	0.508

Supplemental Table S9: CRP and A/G ratio at endpoint are best at discriminating between asymptomatic animals and animals with TB when taking only endpoint measurements into account. We performed similar analyses as in **Supplemental Table S5** except that in the analysis we only included endpoint features.

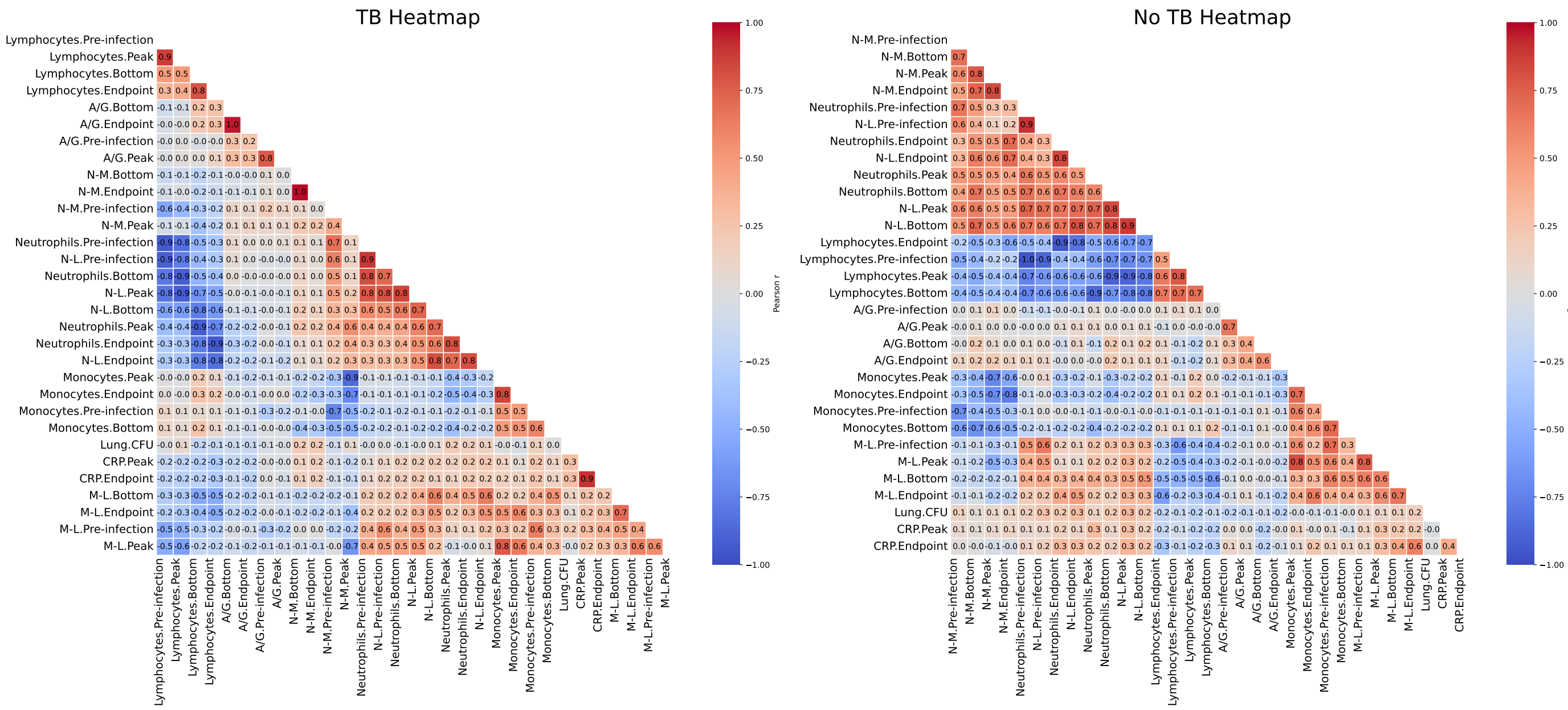

Supplemental Figure S1: Correlation between individual features. We computed pairwise Pearson correlations among all features and visualized them as a heatmap (**A**). To better illustrate groups of correlated variables, we reordered the features using Ward's hierarchical clustering (**B**). Colors range from red (positive correlation) to blue (negative correlation).

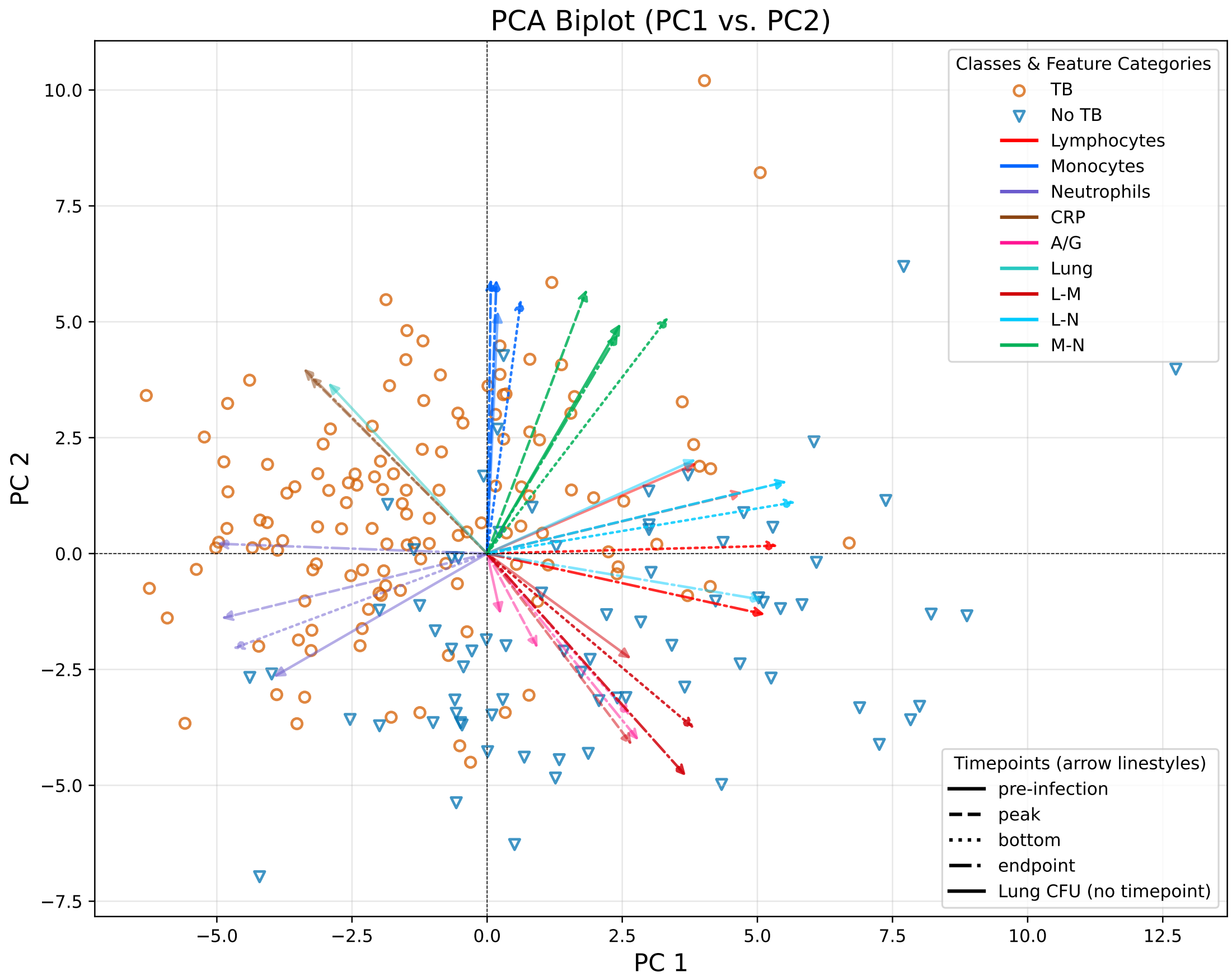

Supplemental Figure S2: Principal component analysis of all features. We applied principal component analysis (PCA) to the full feature set and plotted the first two principal components. Points represent individual animals, colored by disease state (orange = Active disease, blue = asymptomatic). Arrows represent feature loadings, with direction indicating correlation with the principal components and length proportional to loading magnitude. Feature vectors are colored by biological category (e.g., pink = A/G, red = CRP, purple = CFUs, etc.) to aid interpretation.

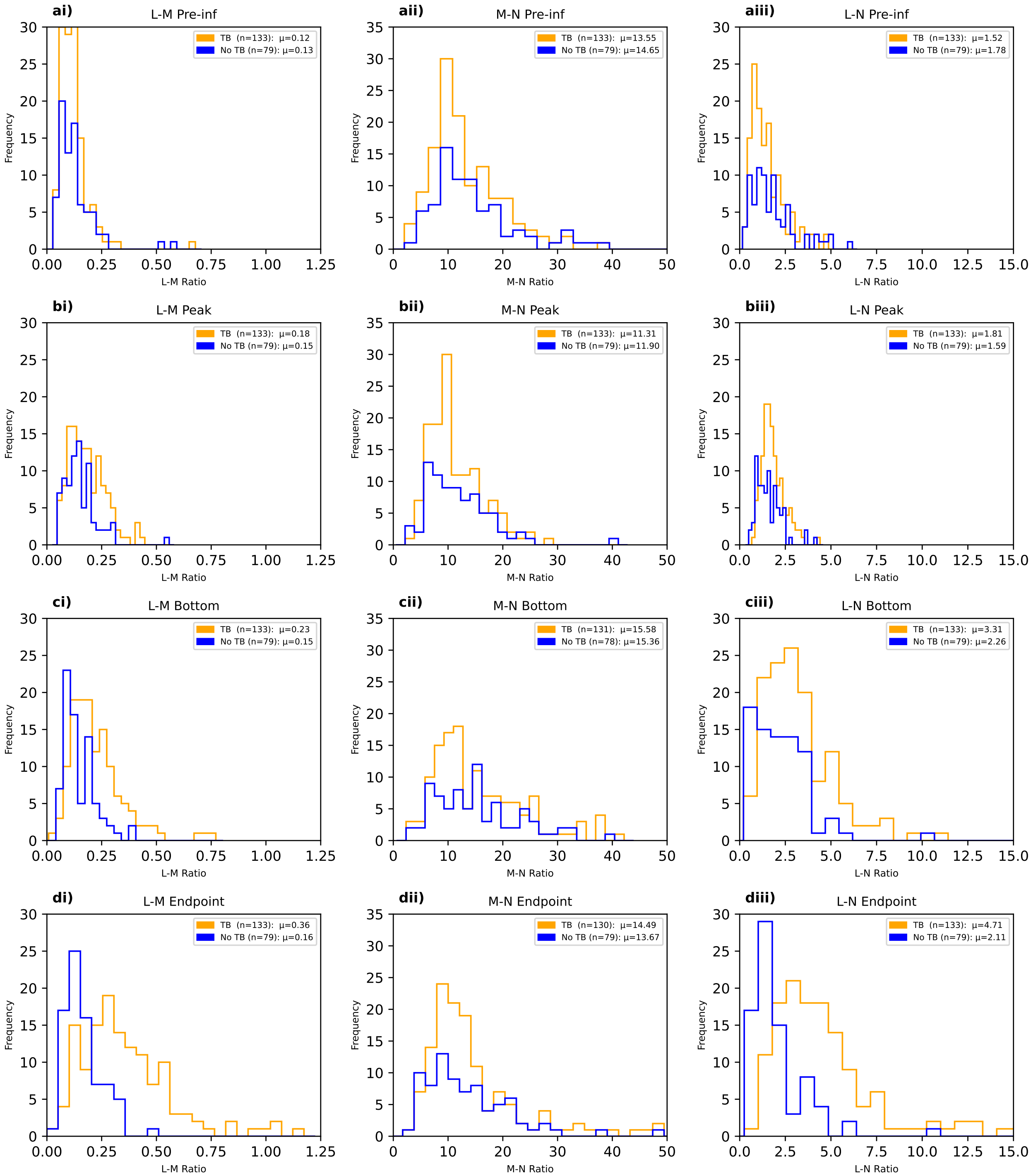

Supplemental Figure S3: Ratio of blood leukocytes do not show a clear separation between animals progressing to active disease vs. asymptomatic animals. We show histograms for the ratios of cell features from this analysis for different time points after infection (**a)**pre-infection, **b)** peak, **c)** bottom, and **d)**endpoint) including (**i**) monocyte to lymphocyte ratio (M-L), (**ii**) neutrophil to lymphocyte ratio (N-L), (**iii**) monocyte to lymphocyte ratio (M-L), Orange histograms are for animals with active disease and blue histograms are for asymptomatic animals.

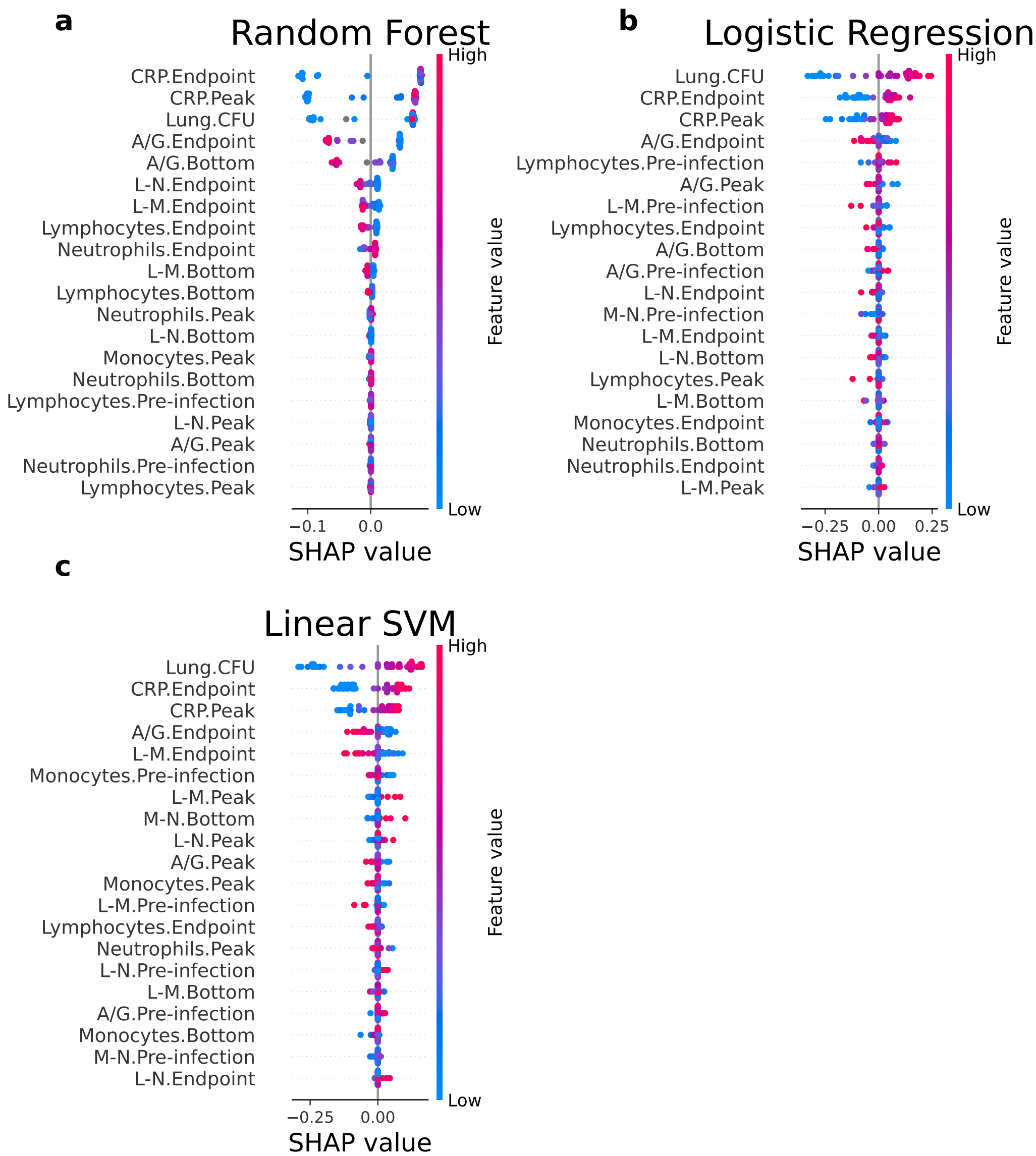

Supplemental Figure S4: Across RF, LR, and linear SVM, a small, repeatable set of features dominates class discrimination when using all features. Each panel is a SHAP summary plot showing per-subject contributions: points are subjects; horizontal position is the SHAP value (impact on the model output); color encodes feature value (blue = low, red = high). Features are ordered by mean absolute SHAP, so higher rows contribute more on average; vertical spread reflects subject-level heterogeneity. We show three models to demonstrate robustness of effect patterns to inductive bias—TreeSHAP for Random Forest (a), KernelSHAP for Logistic Regression (b), and KernelSHAP for linear SVM (c)—providing interpretability that complements permutation-ΔAUC rankings and supports clinical plausibility.

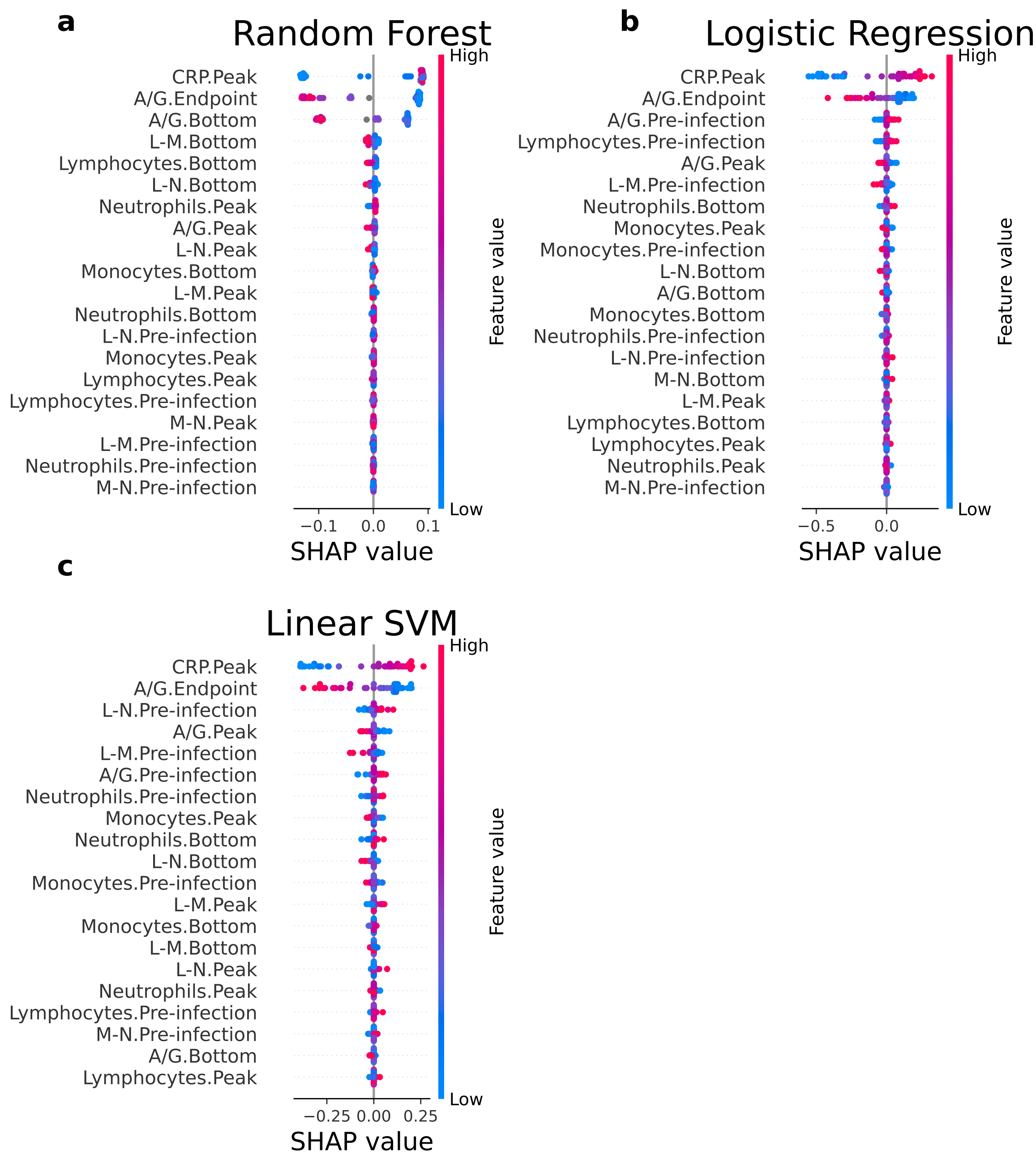

Supplemental Figure S5: Across RF, LR, and linear SVM, a small, repeatable set of features dominates class discrimination when excluding endpoint measurements. Similar analysis as in **Supplemental Figure** S4 except excluding endpoint measurements.

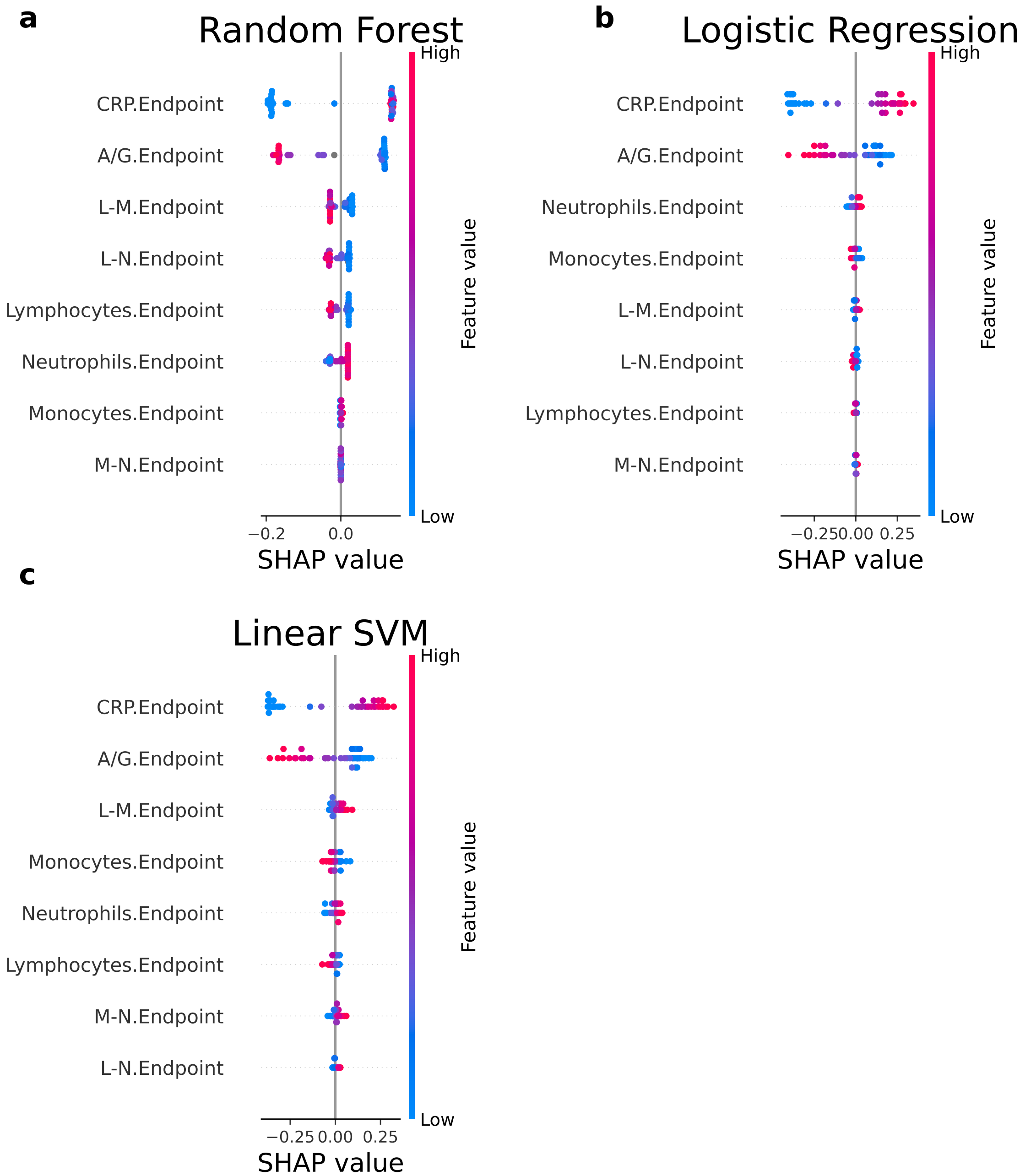

Supplemental Figure S6: Across RF, LR, and linear SVM, a small, repeatable set of features dominates class discrimination when only including endpoint measurements. Similar analysis as in **Supplemental Figure S4** except only including endpoint measurements.

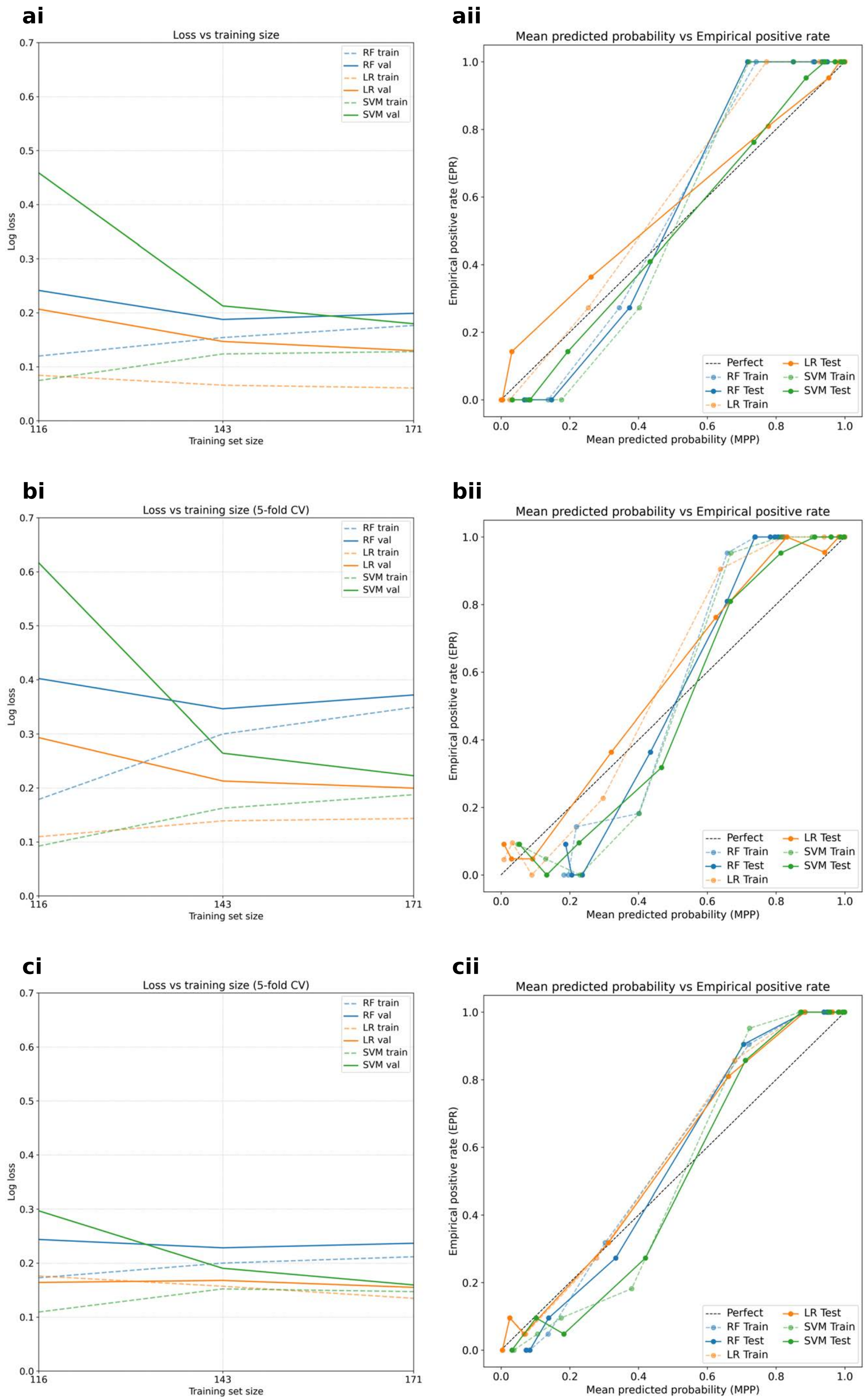

Supplemental Figure S7: Calibration analyses confirm consistent performance of best models across evaluation metrics. (i) We plot log-loss (lower is better) against training set size, showing both training and validation curves. (ii) Calibration plots comparing mean predicted probability (MPP) to empirical positive rate (EPR) indicate close alignment between predicted and observed outcomes. We show results for the full model that included all features (a), model that excludes endpoints (b), or the model that only includes endpoints (c, see **Figure 3** for more detail).

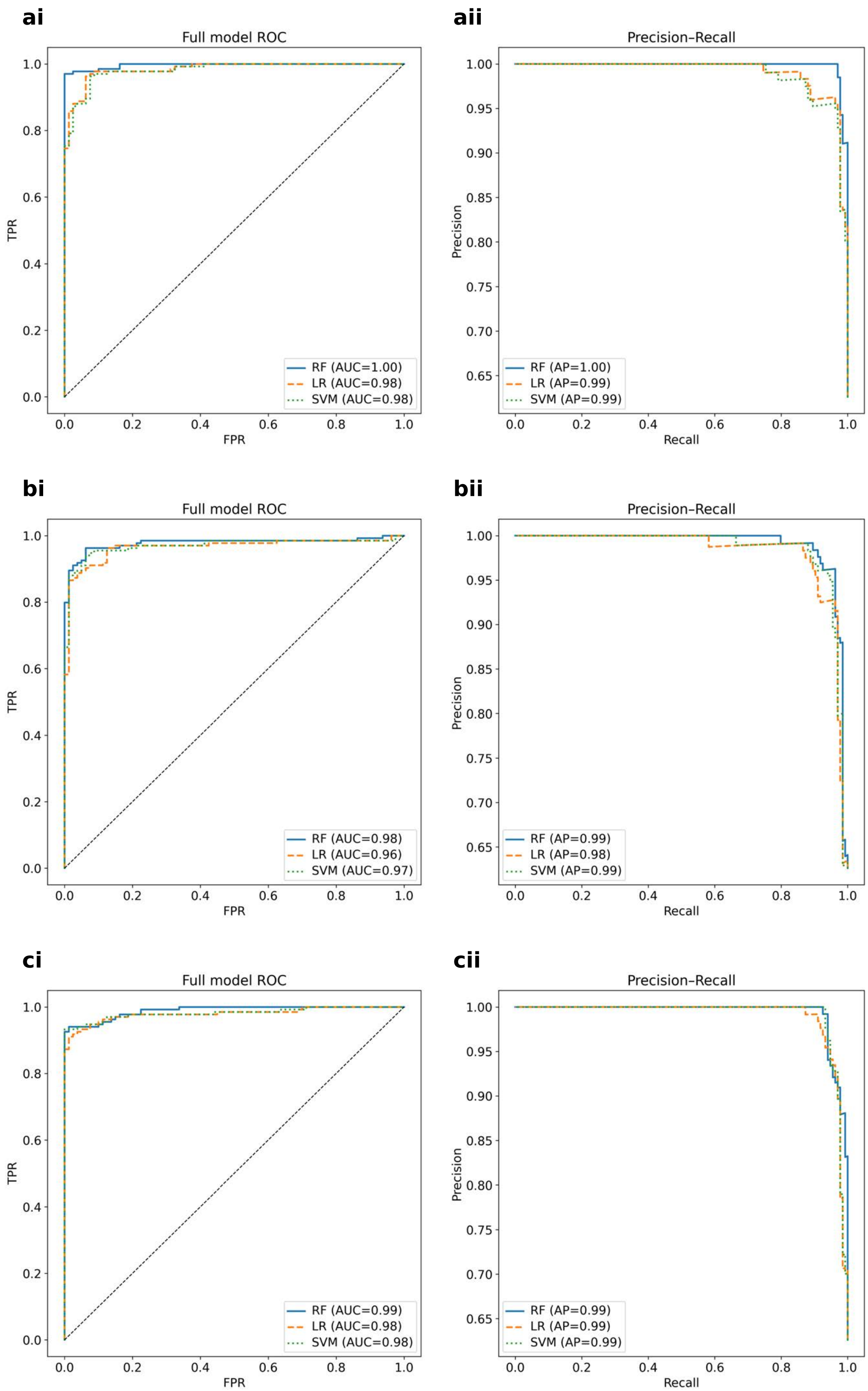

Supplemental Figure S8: Calibration analyses confirm consistent performance of best models across evaluation metrics. (i) Receiver operating characteristic (ROC) curves show high discrimination ability for all models. The curves show the models trained on the full feature set. Since the models were trained on all of the features, we see that the models have very high AUC values, indicating strong performance in distinguishing between the two classes. (ii) Precision–Recall curves highlight reliable precision across recall values. We see that all models maintain high precision even at higher recall levels. We show results for the full model that included all features (a), model that excludes endpoints (b), or the model that only includes endpoints (c, see **Figure 3** for more detail).

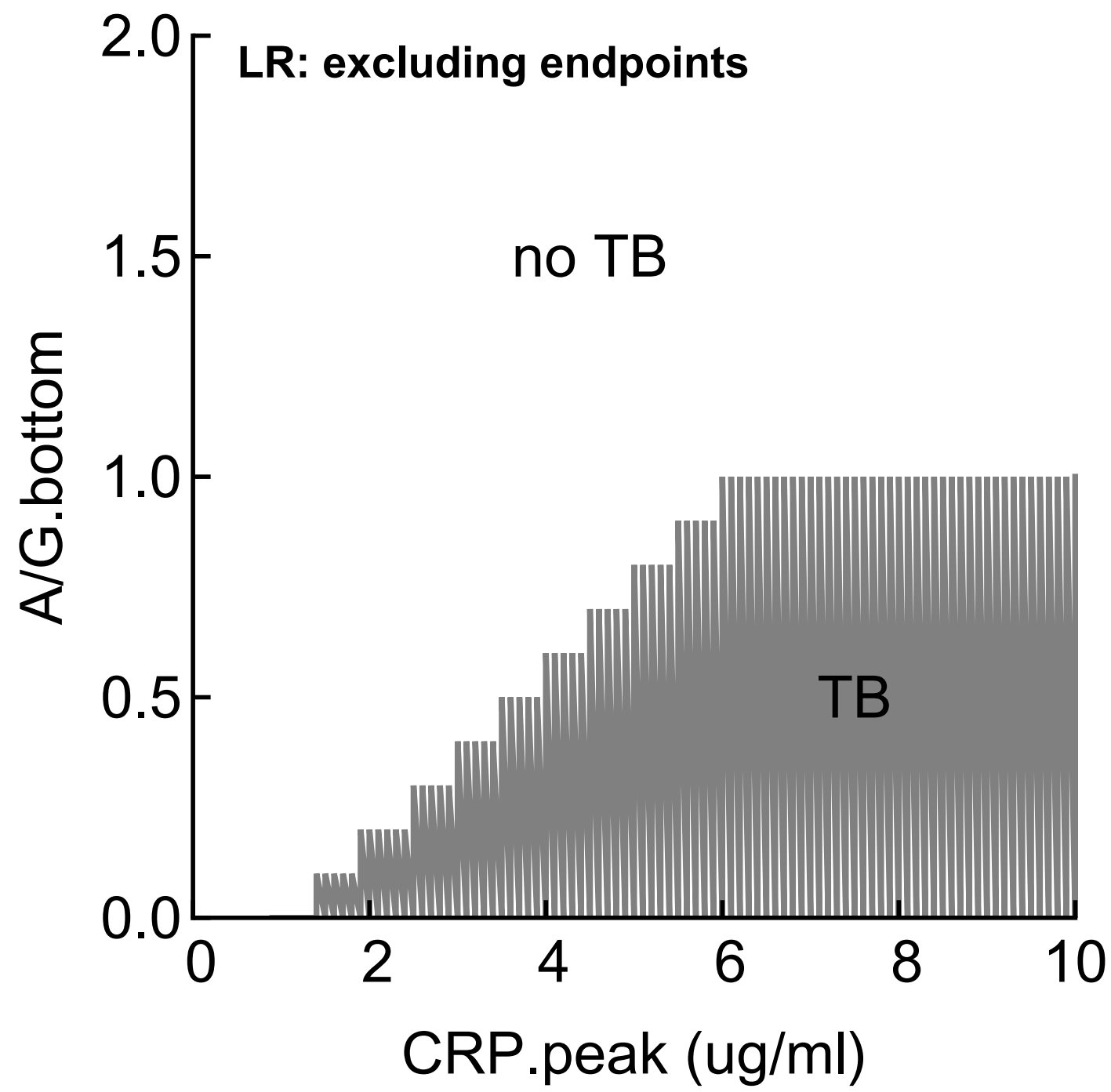

Supplemental Figure S9: LR-based analysis of data that exclude endpoint measurements suggest feature values defining active disease. We used our trained LR model to predict disease status for a range of CRP and A/G ratio values; shaded area defines values for CRP and A/G ratio that correspond to animals with active (symptomatic) disease.
