# Supplementary material for: Machine learning-based feature selection suggests circulating proteins as best at predicting and diagnosing tuberculosis in Mycobacterium tuberculosis-exposed rhesus macaques
Source: Front Cell Infect Microbiol. 2026 Aug 6;16:1880496. doi: 10.3389/fcimb.2026.1880496 (PMC13489891; doi:10.3389/fcimb.2026.1880496)
Supplement: Supplementary file 2 [file DataSheet2.zip › Final_TB_diagram-ver2.pdf]

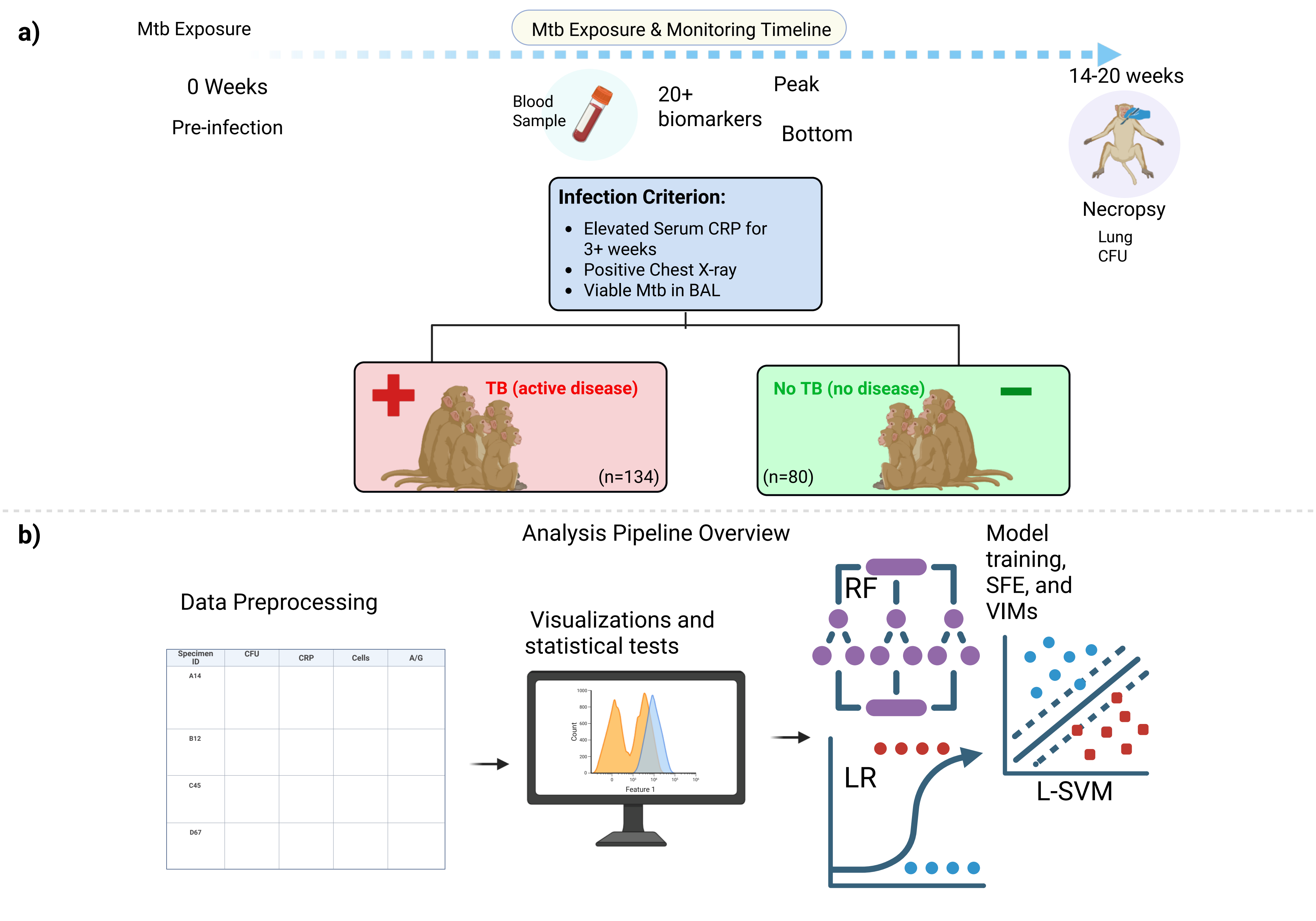

a)
Mtb Exposure
Mtb Exposure & Monitoring Timeline
0 Weeks
Pre-infection
Blood Sample
20+ biomarkers
Peak
Bottom
14-20 weeks
Necropsy
Lung CFU
Infection Criterion:
Elevated Serum CRP for 3+ weeks
Positive Chest X-ray
Viable Mtb in BAL
TB (active disease)
(n=134)
No TB (no disease)
(n=80)
b)
Analysis Pipeline Overview
Data Preprocessing
Specimen ID
CFU
CRP
Cells
A/G
A14
B12
C45
D67
Visualizations and statistical tests
Count
Feature 1
RF
LR
Model training, SFE, and VIMs
L-SVM
