# Supplementary material for: Machine learning-based feature selection suggests circulating proteins as best at predicting and diagnosing tuberculosis in Mycobacterium tuberculosis-exposed rhesus macaques
Source: Front Cell Infect Microbiol. 2026 Aug 6;16:1880496. doi: 10.3389/fcimb.2026.1880496 (PMC13489891; doi:10.3389/fcimb.2026.1880496)
Supplement: Supplementary file 2 [file DataSheet2.zip › tb_stephistograms_w-ratios-new3-AI.pdf]

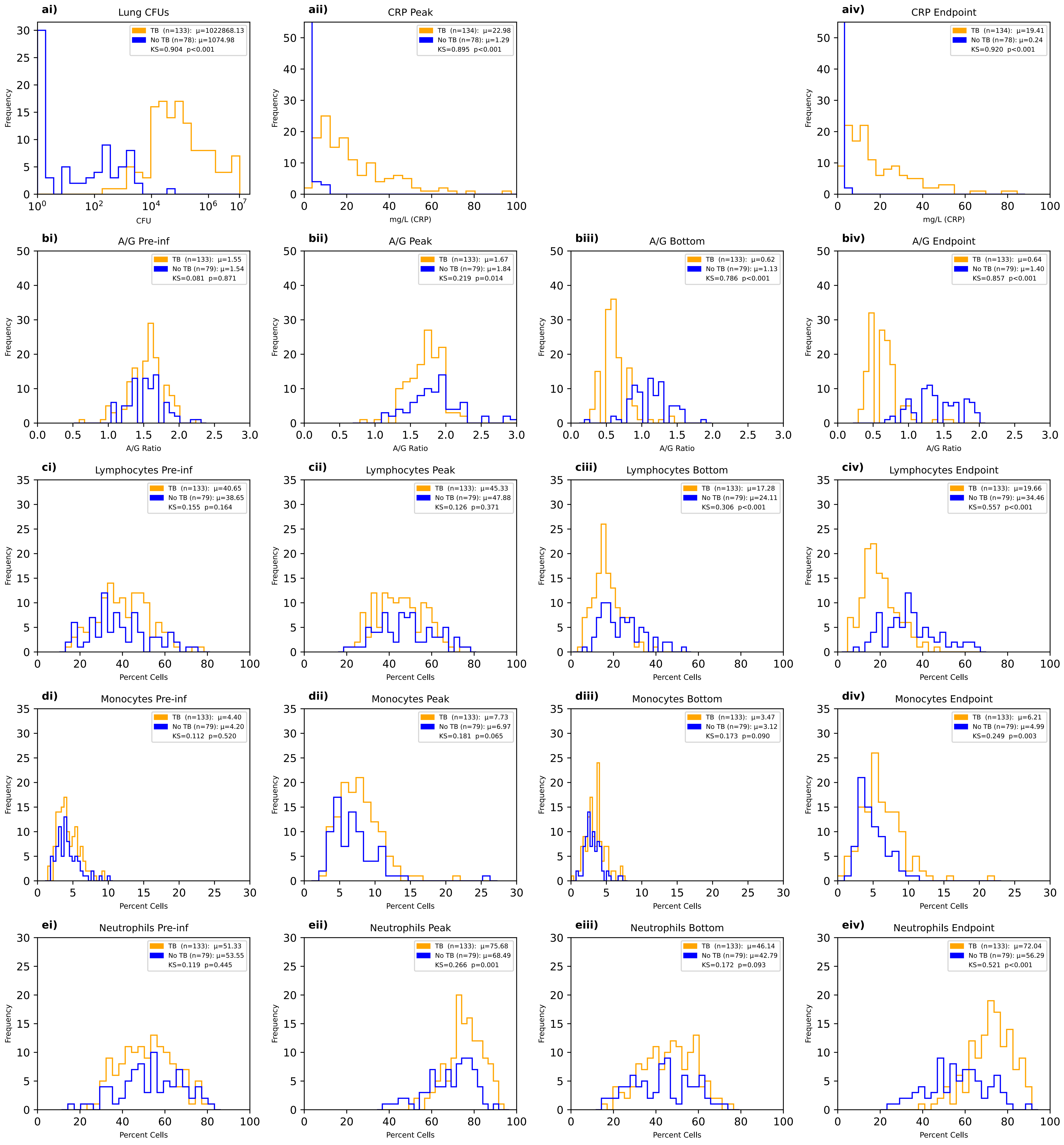

ai) Lung CFUs
TB (n=133): μ=1022868.13
No TB (n=78): μ=1074.98
KS=0.904 p<0.001
Frequency
CFU
aii) CRP Peak
TB (n=134): μ=22.98
No TB (n=78): μ=1.29
KS=0.895 p<0.001
mg/L (CRP)
aiv) CRP Endpoint
TB (n=134): μ=19.41
No TB (n=78): μ=0.24
KS=0.920 p<0.001
mg/L (CRP)
bi) A/G Pre-inf
TB (n=133): μ=1.55
No TB (n=79): μ=1.54
KS=0.081 p=0.871
A/G Ratio
bii) A/G Peak
TB (n=133): μ=1.67
No TB (n=79): μ=1.84
KS=0.219 p=0.014
A/G Ratio
biii) A/G Bottom
TB (n=133): μ=0.62
No TB (n=79): μ=1.13
KS=0.786 p<0.001
A/G Ratio
biv) A/G Endpoint
TB (n=133): μ=0.64
No TB (n=79): μ=1.40
KS=0.857 p<0.001
A/G Ratio
ci) Lymphocytes Pre-inf
TB (n=133): μ=40.65
No TB (n=79): μ=38.65
KS=0.155 p=0.164
Percent Cells
cii) Lymphocytes Peak
TB (n=133): μ=45.33
No TB (n=79): μ=47.88
KS=0.126 p=0.371
Percent Cells
ciii) Lymphocytes Bottom
TB (n=133): μ=17.28
No TB (n=79): μ=24.11
KS=0.306 p<0.001
Percent Cells
civ) Lymphocytes Endpoint
TB (n=133): μ=19.66
No TB (n=79): μ=34.46
KS=0.557 p<0.001
Percent Cells
di) Monocytes Pre-inf
TB (n=133): μ=4.40
No TB (n=79): μ=4.20
KS=0.112 p=0.520
Percent Cells
dii) Monocytes Peak
TB (n=133): μ=7.73
No TB (n=79): μ=6.97
KS=0.181 p=0.065
Percent Cells
diii) Monocytes Bottom
TB (n=133): μ=3.47
No TB (n=79): μ=3.12
KS=0.173 p=0.090
Percent Cells
div) Monocytes Endpoint
TB (n=133): μ=6.21
No TB (n=79): μ=4.99
KS=0.249 p=0.003
Percent Cells
ei) Neutrophils Pre-inf
TB (n=133): μ=51.33
No TB (n=79): μ=53.55
KS=0.119 p=0.445
Percent Cells
eii) Neutrophils Peak
TB (n=133): μ=75.68
No TB (n=79): μ=68.49
KS=0.266 p=0.001
Percent Cells
eiii) Neutrophils Bottom
TB (n=133): μ=46.14
No TB (n=79): μ=42.79
KS=0.172 p=0.093
Percent Cells
eiv) Neutrophils Endpoint
TB (n=133): μ=72.04
No TB (n=79): μ=56.29
KS=0.521 p<0.001
Percent Cells
