# Supplementary material for: Machine learning-based feature selection suggests circulating proteins as best at predicting and diagnosing tuberculosis in Mycobacterium tuberculosis-exposed rhesus macaques
Source: Front Cell Infect Microbiol. 2026 Aug 6;16:1880496. doi: 10.3389/fcimb.2026.1880496 (PMC13489891; doi:10.3389/fcimb.2026.1880496)
Supplement: Supplementary file 2 [file DataSheet2.zip › sfe_roc_stacked-new3.pdf]

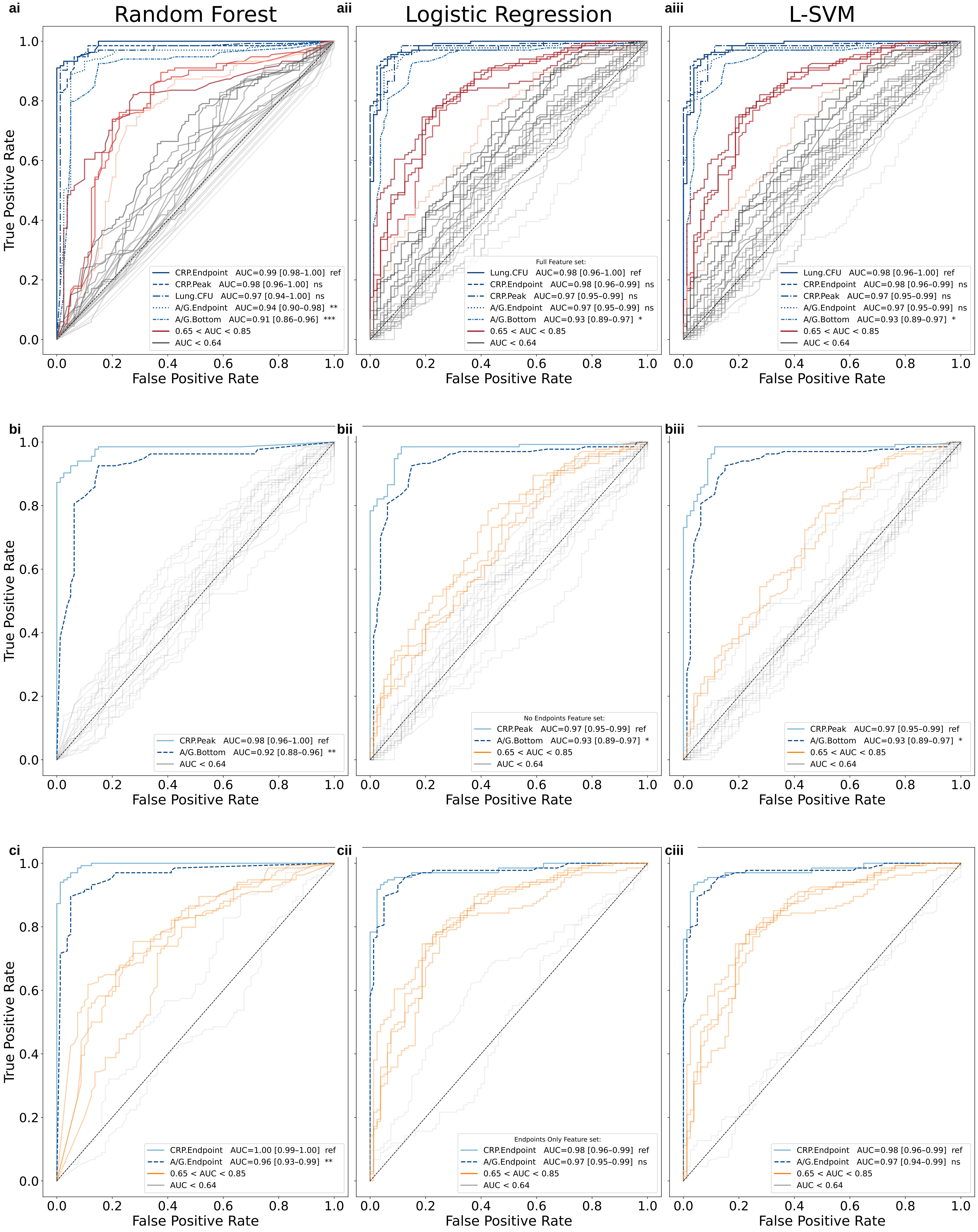

ai
Random Forest
aii
Logistic Regression
aiii
L-SVM
True Positive Rate
False Positive Rate
CRP.Endpoint AUC=0.99 [0.98–1.00] ref
CRP.Peak AUC=0.98 [0.96–1.00] ns
Lung.CFU AUC=0.97 [0.94–1.00] ns
A/G.Endpoint AUC=0.94 [0.90–0.98] **
A/G.Bottom AUC=0.91 [0.86–0.96] ***
0.65 < AUC < 0.85
AUC < 0.64
Full Feature set:
Lung.CFU AUC=0.98 [0.96–1.00] ref
CRP.Endpoint AUC=0.98 [0.96–0.99] ns
CRP.Peak AUC=0.97 [0.95–0.99] ns
A/G.Endpoint AUC=0.97 [0.95–0.99] ns
A/G.Bottom AUC=0.93 [0.89–0.97] *
0.65 < AUC < 0.85
AUC < 0.64
Lung.CFU AUC=0.98 [0.96–1.00] ref
CRP.Endpoint AUC=0.98 [0.96–0.99] ns
CRP.Peak AUC=0.97 [0.95–0.99] ns
A/G.Endpoint AUC=0.97 [0.95–0.99] ns
A/G.Bottom AUC=0.93 [0.89–0.97] *
0.65 < AUC < 0.85
AUC < 0.64
bi
bii
biii
CRP.Peak AUC=0.98 [0.96–1.00] ref
A/G.Bottom AUC=0.92 [0.88–0.96] **
AUC < 0.64
No Endpoints Feature set:
CRP.Peak AUC=0.97 [0.95–0.99] ref
A/G.Bottom AUC=0.93 [0.89–0.97] *
0.65 < AUC < 0.85
AUC < 0.64
CRP.Peak AUC=0.97 [0.95–0.99] ref
A/G.Bottom AUC=0.93 [0.89–0.97] *
0.65 < AUC < 0.85
AUC < 0.64
ci
cii
ciii
CRP.Endpoint AUC=1.00 [0.99–1.00] ref
A/G.Endpoint AUC=0.96 [0.93–0.99] **
0.65 < AUC < 0.85
AUC < 0.64
Endpoints Only Feature set:
CRP.Endpoint AUC=0.98 [0.96–0.99] ref
A/G.Endpoint AUC=0.97 [0.95–0.99] ns
0.65 < AUC < 0.85
AUC < 0.64
CRP.Endpoint AUC=0.98 [0.96–0.99] ref
A/G.Endpoint AUC=0.97 [0.94–0.99] ns
0.65 < AUC < 0.85
AUC < 0.64
