# Supplementary material for: Machine learning-based feature selection suggests circulating proteins as best at predicting and diagnosing tuberculosis in Mycobacterium tuberculosis-exposed rhesus macaques
Source: Front Cell Infect Microbiol. 2026 Aug 6;16:1880496. doi: 10.3389/fcimb.2026.1880496 (PMC13489891; doi:10.3389/fcimb.2026.1880496)
Supplement: Supplementary file 2 [file DataSheet2.zip › full_models_stacked-new2.pdf]

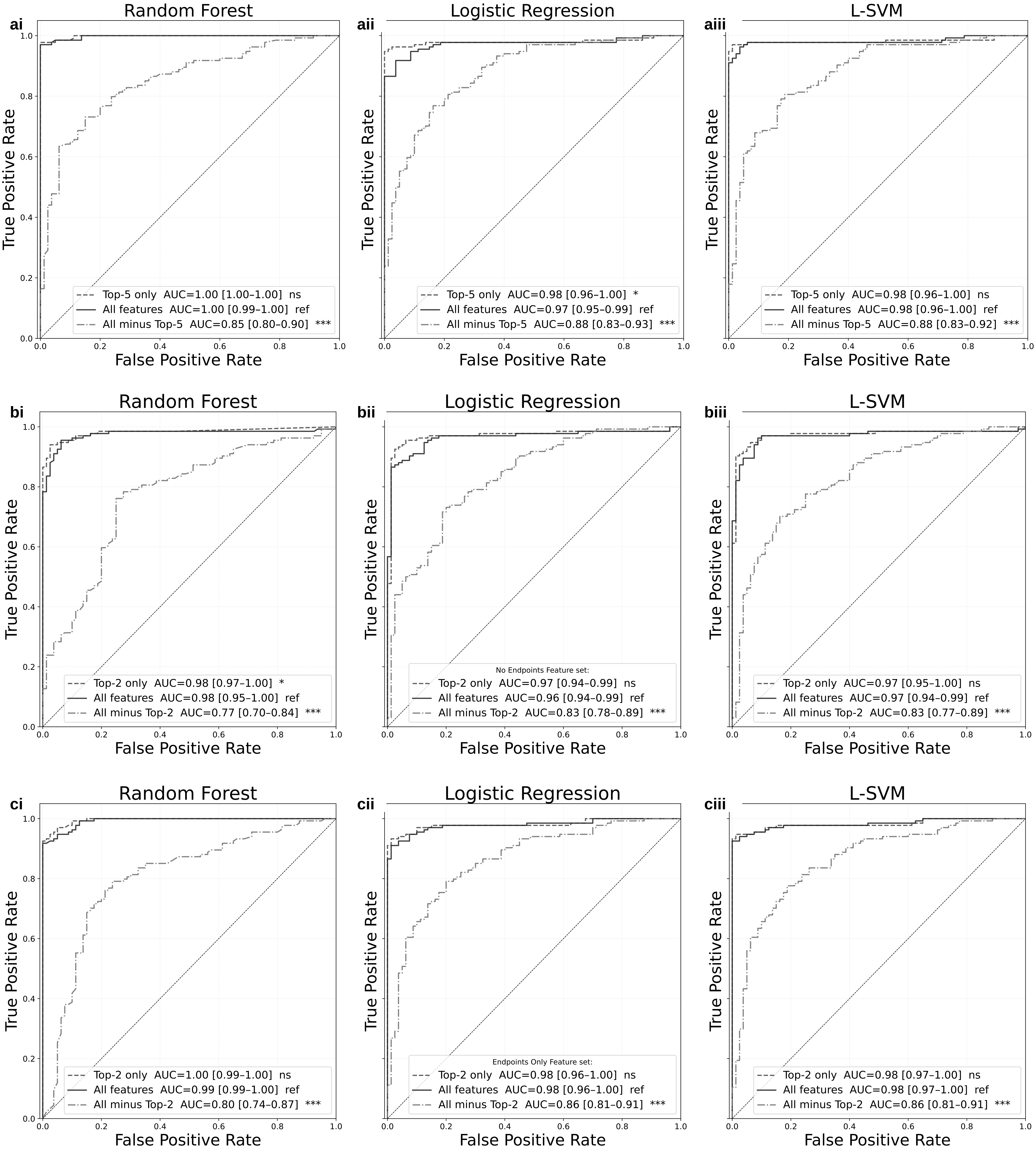
ai
Random Forest
True Positive Rate
False Positive Rate
Top-5 only AUC=1.00 [1.00–1.00] ns
All features AUC=1.00 [0.99–1.00] ref
All minus Top-5 AUC=0.85 [0.80–0.90] ***
aii
Logistic Regression
True Positive Rate
False Positive Rate
Top-5 only AUC=0.98 [0.96–1.00] *
All features AUC=0.97 [0.95–0.99] ref
All minus Top-5 AUC=0.88 [0.83–0.93] ***
aiii
L-SVM
True Positive Rate
False Positive Rate
Top-5 only AUC=0.98 [0.96–1.00] ns
All features AUC=0.98 [0.96–1.00] ref
All minus Top-5 AUC=0.88 [0.83–0.92] ***
bi
Random Forest
True Positive Rate
False Positive Rate
Top-2 only AUC=0.98 [0.97–1.00] *
All features AUC=0.98 [0.95–1.00] ref
All minus Top-2 AUC=0.77 [0.70–0.84] ***
bii
Logistic Regression
True Positive Rate
False Positive Rate
No Endpoints Feature set:
Top-2 only AUC=0.97 [0.94–0.99] ns
All features AUC=0.96 [0.94–0.99] ref
All minus Top-2 AUC=0.83 [0.78–0.89] ***
biii
L-SVM
True Positive Rate
False Positive Rate
Top-2 only AUC=0.97 [0.95–1.00] ns
All features AUC=0.97 [0.94–0.99] ref
All minus Top-2 AUC=0.83 [0.77–0.89] ***
ci
Random Forest
True Positive Rate
False Positive Rate
Top-2 only AUC=1.00 [0.99–1.00] ns
All features AUC=0.99 [0.99–1.00] ref
All minus Top-2 AUC=0.80 [0.74–0.87] ***
cii
Logistic Regression
True Positive Rate
False Positive Rate
Endpoints Only Feature set:
Top-2 only AUC=0.98 [0.96–1.00] ns
All features AUC=0.98 [0.96–1.00] ref
All minus Top-2 AUC=0.86 [0.81–0.91] ***
ciii
L-SVM
True Positive Rate
False Positive Rate
Top-2 only AUC=0.98 [0.97–1.00] ns
All features AUC=0.98 [0.97–1.00] ref
All minus Top-2 AUC=0.86 [0.81–0.91] ***
